# 3D dendritic spines shape descriptors for efficient classification and morphology analysis in control and Alzheimer’s disease modeling neurons

**DOI:** 10.1093/bioinformatics/btag025

**Published:** 2026-01-20

**Authors:** Daria Smirnova, Anita Ustinova, Viacheslav Chukanov, Ekaterina Pchitskaya

**Affiliations:** Laboratory of Biomedical Imaging and Data Analysis, Institute of Biomedical Systems and Biotechnology, Peter the Great St. Petersburg Polytechnic University, Khlopina St. 11, St. Petersburg, 194021, Russia; Laboratory of Biomedical Imaging and Data Analysis, Institute of Biomedical Systems and Biotechnology, Peter the Great St. Petersburg Polytechnic University, Khlopina St. 11, St. Petersburg, 194021, Russia; Laboratory of Biomedical Imaging and Data Analysis, Institute of Biomedical Systems and Biotechnology, Peter the Great St. Petersburg Polytechnic University, Khlopina St. 11, St. Petersburg, 194021, Russia; Department of Applied Mathematics, Peter the Great St. Petersburg Polytechnic University, Polytechnicheskaya St. 29, St. Petersburg, 195251, Russia; Laboratory of Biomedical Imaging and Data Analysis, Institute of Biomedical Systems and Biotechnology, Peter the Great St. Petersburg Polytechnic University, Khlopina St. 11, St. Petersburg, 194021, Russia; Laboratory of Molecular Neurodegeneration, Institute of Biomedical Systems and Biotechnology, Peter the Great St. Petersburg Polytechnic University, Khlopina St. 11, St. Petersburg, 194021, Russia

## Abstract

**Motivation:**

Dendritic spines, postsynaptic structures characterized by their complex shapes, provide the essential structural foundation for synaptic function. Their shape is dynamic, undergoing alterations in various conditions, notably during neurodegenerative disorders like Alzheimer’s disease. The dramatically increasing prevalence of such diseases highlights an urgent need for effective treatments. A key strategy in developing these treatments involves evaluating how dendritic spine morphology responds to potential therapeutic compounds. Although a link between spine shape and function is recognized, its precise nature is still not fully elucidated. Consequently, advancing our understanding of dendritic spines in both health and disease necessitates the urgent development of more effective methods for assessing their morphology.

**Results:**

This study introduces qualitatively new 3D dendritic shape descriptors based on spherical harmonics and Zernike moments and proposes a bases on them clustering approach for grouping dendritic spines with similar shapes applied to 3D polygonal spines meshes acquired from Z-stack dendrite images. By integrating these methods, we achieve improved differentiation between normal and pathological spines represented by the Alzheimer’s disease in vitro model, offering a more precise representation of morphological diversity. Additionally, the proposed spherical harmonics approach enables dendritic spine reconstruction from vector-based shape representations, providing a novel tool for studying structural changes associated with neurodegeneration and possibilities for synthetic dendritic spines dataset generation.

**Availability and implementation:**

The software used for experiments is public and available at https://github.com/Biomed-imaging-lab/SpineTool with the DOI: 10.5281/zenodo.17359066. Descriptors codebase is available at https://github.com/Biomed-imaging-lab/Spine-Shape-Descriptors with the DOI: 10.5281/zenodo.17302859.

## 1 Introduction

Dendritic spines (spines) are small protrusions from dendrites form functional connections with axons of other neurons (Smith *et al.* 2014). Spines are crucial for memory formation and storage, playing a key role in synaptic potentiation and depression (Bailey *et al.* 2015). Learning and memory consolidation are closely tied to the remodeling or elimination of existing dendritic spines and the creation of new ones, which enhances the efficiency of information transfer between neurons (Chidambaram *et al.* 2019, Stein and Zito 2019). Their size and shape constantly changing during normal brain function (Steffens *et al.* 2021), and this dynamic nature is supported by a complex network of signaling molecules and cascades (Nakahata and Yasuda 2018). Dendritic spines exhibit changes in response to various stimuli, including drug administration (Barrientos *et al.* 2018), hypoxia (Saraceno *et al.* 2012), environmental changes (Ashokan *et al.* 2018), and also various neurodevelopmental, neurodegenerative, and psychiatric conditions (Penzes *et al.* 2011). For instance, Alzheimer’s disease (AD) is associated with dendritic spine shrinkage and elimination in the hippocampus and cortex, potentially occurring before clinical symptoms such as cognitive decline and memory dysfunction appear (Boros *et al.* 2017). Normalization of dendritic spines structure may be the one of the therapeutic strategies and a valuable outcome in preclinical drug testing for Alzheimer’s and other neurodegenerative diseases.

Dendritic spines display considerable structural diversity, with sizes that differ greatly and shapes varying from elongated protrusions to rounded, mushroom-like forms, forming a continuum of morphological kinds (Loewenstein *et al.* 2011, Ofer *et al.* 2022). Dendritic spine morphology is usually connected with their functionality (Arellano *et al.* 2007), so it is essential to separate different morphological shapes and features of spines. Traditionally, various research studies distinguish between three to five morphological classes, with the most commonly accepted classification categorizing dendritic spines into mushroom, thin, and stubby types (Bourne and Harris 2008, Rodriguez *et al.* 2008). Some researchers also distinguish filopodia as a separate category (Yuste and Bonhoeffer 2001). The limitations of the classification approach lie in the subjective determination of morphological groups, which can lead to inconsistencies (Pchitskaya *et al.* 2023).

In recent years, the concept of a continuous spectrum of dendritic spine morphologies has gained increasing recognition. Studies using in vitro and in vivo live microscopy suggest that dendritic spine shapes exist along a continuum rather than discrete morphological categories (Arellano *et al.* 2007, Bhatt *et al.* 2009, Loewenstein *et al.* 2011, Ofer *et al.* 2021, Ofer *et al.* 2022). Several clustering methods and criteria for determining the optimal number of clusters have been previously applied, and the question of the most appropriate method remains open (Bokota *et al.* 2016, Ghani *et al.* 2016, Luengo-Sanchez *et al.* 2018, Pchitskaya and Bezprozvanny 2020, Pchitskaya *et al.* 2023). Clustering is also used in research as an auxiliary tool for analyzing morphological changes (Ferreira *et al.* 2024). In this study, we evaluate clustering approach on dataset of control and AD modeling dendritic spines. This approach aims to enhance sensitivity in distinguishing between normal and pathological spines, potentially providing more insightful results than traditional classification methods.

For the analysis of dendritic spine morphology, whether through classification or clustering, it is essential to measure their shape. Initially, shape descriptors were proposed as scalar metrics (Bokota *et al.* 2016, Kashiwagi *et al.* 2019). The length of the spine, volume, average head width of the spine, mean angle between the axis of the spine and its vertices and other scalar metrics only indirectly allow for an assessment of the spine’s actual shape, most of this descriptors being more indicative of spine’s size. Thus, scalar descriptors may not capture the full complexity of spine morphology, limiting the accuracy and reliability of the analysis. Developing chord length distribution histogram metric (CLDH) was the first step in utilizing advanced methods (Pchitskaya *et al.* 2023). CLDH showed more accurate in clustering task morphology that has invariant to rotation. But a possible limitation of CLDH is lacking local location-based specificity and the non-surjectivity of the representation, which may lead to identical representations of different shapes.

In this study, we go further with usage of complex shape descriptor and propose 3D dendritic spine shape encoding methods based on spherical harmonics (SphHarm) and Light Field (LF), that provide a direct description of the spine’s shape. Both approaches have shown strong performance in engineering shape analysis. In biology, spherical harmonics have been applied to cell shape dynamics (Medyukhina *et al.* 2020) and caudal brain region morphology (Styner *et al.* 2006), while a Light Field component—Zernike moments—improved mammogram tumor classification (Tahmasbi *et al.* 2011, Sharma and Khanna 2015).

By comparing these novel 3D encoding methods for analyzing dendritic spine morphology to classical features using clustering approaches, we demonstrate their enhanced sensitivity to morphological variability by separating control spines and spines with the AD cell model across clusters. In addition to clustering, we also examine their performance in the classification task, which remains a relevant and widely adopted method in neuroscientific research. Moreover, the spherical harmonics descriptor enables the reconstruction of realistic dendritic spine shapes from vector data. We developed a technique for spine shape reconstruction and compared it with an existing method based on ellipse approximation (Luengo-Sanchez *et al.* 2018), showing superior results. This advancement makes it possible to model spine shapes and generate synthetic datasets of dendrites and dendritic spines with complex shape descriptors.

## 2 Materials and methods

### 2.1 Dataset preparation and analysis

**Figure 1:**
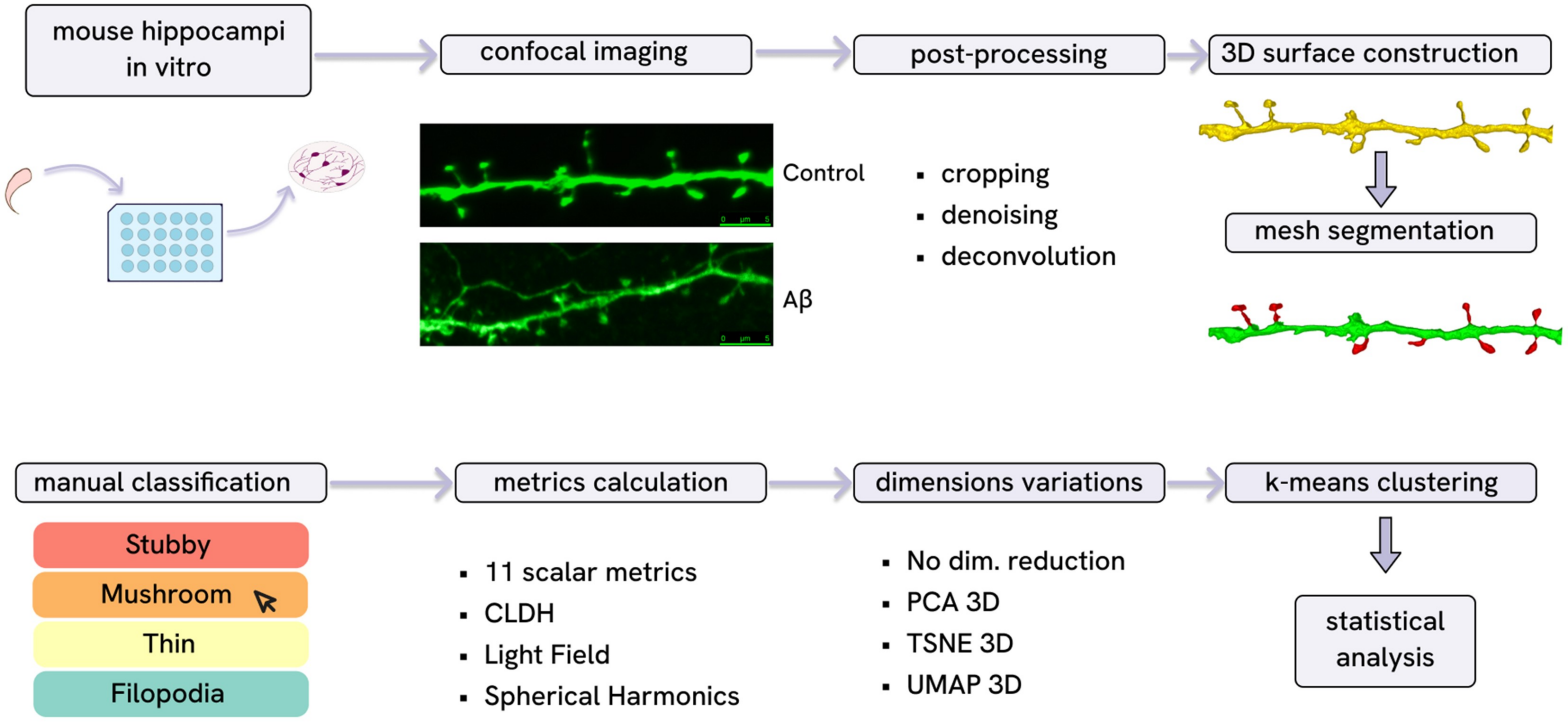
Dendritic spines analysis pipeline using SpineTool software including biological data acquisition, imaging and image post-processing, obtaining polygonal meshes of the dendrite and spines, metrics calculation, classification and clustering.

The experimental pipeline is illustrated in Fig. 1 (Ustinova *et al.* 2024). All animal procedures were approved by the Bioethics Committee of the Peter the Great St Petersburg Polytechnic University at St Petersburg, Russia and followed the principles of European convention (Strasbourg, 1986) and the Declaration of International medical association about humane treatment of animals (Helsinki, 1996). Primary hippocampal neuronal cultures of dissociated hippocampal cells were prepared from newborn Albino inbred mice (FVB/NJ), which were obtained from the Jackson Laboratory (Jackson Laboratory, Bar Harbor, ME, USA, strain #001800) and maintained in culture as described previously (Popugaeva *et al.* 2015). Briefly, primary hippocampal neurons were transfected with the pLV-eGFP plasmid for visualization and cultivated in vitro for 16–17 days (control group). A portion of these neurons was exposed to amyloid toxicity conditions (Aβ group) for 72 h prior to fixation to model low amyloid synaptotoxicity, more detailed information is provided in Supplementary Material 1, available as supplementary data at *Bioinformatics* online. Proper description of confocal microscopy, image preprocessing, spines segmentation and analysis in SpineTool (Pchitskaya *et al.* 2023) is also presented in Supplementary Material 1, available as supplementary data at *Bioinformatics* online.

### 2.2 Dendritic spines encoding with 3D shape descriptors

#### 2.2.1 Shape approximation with equation and its accuracy estimation

Instead of using scalar values and statistical samples to characterize the dendritic spines morphology, we propose using an equation-based approach with orthogonal decomposition. Within the context of 3D surfaces, we explore both the surface itself in its 3D representation and certain projections thereof, which encapsulate a 2D perspective. Therefore, two approximation scenarios of the original morphology are used to analyze the morphological features of the dendritic spines: 2D and 3D approaches (for details see Section 1, available as supplementary data at *Bioinformatics* online).

#### 2.2.2 Spine mesh encoding with spherical harmonics

The real-valued spherical harmonic is not invariant to rotation, so to compare, objects must be oriented identically. To use harmonics to encode and then compare spine morphology pairwise within dataset, we algorithmically rotated each spine so that the edges of the joint region were most parallel to the *xz*-axis plane and the largest variance was aligned with the *z*-axis. The alignment with the *xz*-plane was achieved by minimizing the mean angular deviation between the region’s normal vector and the y-axis, while the direction of maximum variance was determined using principal component analysis (PCA).

As the method involves decomposing a spherical function into an orthonormal basis, it is essential to ensure that the encoded object is represented in spherical coordinates, implying that it must exhibit a star-shaped structure. If an object intersects a ray emitted from the origin of the coordinate system multiple times, ambiguity arises. Given that dendritic spines are not inherently spherical surfaces, minimizing such ambiguities is a key objective. In the proposed algorithm, this is achieved through the optimal positioning of the object relative to the center of the spherical coordinate system. To achieve this objective, we introduce “internal center” of the object defined as the point onto object skeleton that is closest point to the object’s center of mass. The “internal center” point is then considered the center of the spherical coordinate system, achieved through a coordinate system transformation. Such an operation is performed on each spine.

To describe spine mesh in spherical coordinates the tabular function for set of uniformly picked coordinates is formed. A tabular function maps a pair of spherical coordinates—azimuthal and zenith angles—to a radius value, where a ray emitted from the center in the direction defined by these angles intersects the described surface. This tabular function serves as the basis for approximating the surface using spherical harmonics. There are various techniques to uniformly sample points on sphere. To sample points we use Monte Carlo algorithm (Dimov *et al.* 2007). Generally, the more sample points provide the more accurate approximation, especially for complex-shaped objects with fine details in local regions. But additional sample points increase the required computational time.

Analytically, the decomposition coefficients are derived by projecting the target spherical function onto the basis functions $Y_{l}^{m}$(θ, ϕ) where *l* is the degree (frequency level) and *m* is the order of the spherical harmonic. The projecting is described as following:


$$a_{lm}=\int_{0}^{2\pi }\int_{0}^{\pi }f(\theta ,\phi )Y_{l}^{m}(\theta ,\phi )\sin\left(\theta \right)d\theta d\phi$$

Numerically, this integral can be approximated by a finite sum:


$$a_{lm}=\frac{4\pi }{\left|S\right|}\sum_{(\theta ,\phi )\in S}f(\theta ,\phi )Y_{l}^{m}(\theta ,\phi ),$$

where (θ, ϕ) is chosen uniformly. So, decomposition coefficients are obtained by evaluating this sum for each of basis functions for choose polynomial degree L. The values of θ, ϕ and f(θ, ϕ) is obtained from a tabular function.

The encoding method parameters are the approximation degree L and sample points number *N*. Both parameters are integer and greater than 1. To determine the optimal values, we performed a grid search over a range of parameter combinations. The parameters corresponding to the onset of both the accuracy plateau and the convergence plateau were considered optimal for our dataset. The detailed analysis of grid search results is provided in Supplementary Material 1, available as supplementary data at *Bioinformatics* online. Based on this analysis, the optimal values were found to be *L* = 10 and *N* = 140 (Fig. 1A, available as supplementary data at *Bioinformatics* online).

#### 2.2.3 Spine shape reconstruction from spherical harmonics decomposition

Approximated mesh reconstruction is performed onto a set of sampled spherical coordinates θ and ϕ. For mesh initialization, an icosphere is used. After approximating the unit sphere by icosphere with a sufficient number of subdivisions for convenient visual assessment, each mesh vertex V_θϕρ_ is transformed to match approximated surface. Here, *ρ* denotes the radial distance from the sphere center. The transformation replaces the vertex coordinates such that the corresponding azimuthal and zenith angles are assigned a radius value computed as the weighted sum of the decomposition basis functions, where the weights are given by the decomposition coefficients:


$$V_{\theta \phi \rho }=(\theta ,\phi ,\sum_{l<L,|m|\leq l}a_{lm}Y_{l}^{m}(\theta ,\phi ))$$

The formed mesh is considered as approximation mesh and could be used for approximation accuracy calculation and visual comparison and evaluation.

#### 2.2.4 Spine mesh encoding with light field descriptor

To represent 3D object with 2D descriptor spine orientation technique described in spherical harmonics mesh preprocessing is also used. Spine mesh then is circled with sphere and observation points on it was chosen: A (0, 0, 2*r*), B (0, π/2, 2*r*), C (π/2, π/2, 2*r*), D (π/3, π/3, 2r) and E (π/3, 2π/3, 2*r*) where r is the maximum radius of the spine vertices in spherical coordinates system. The first three points correspond to the principal orthogonal projections of the object, which are widely used in engineering applications and provide an effective representation of the 3D structure. The other two points were selected at different diagonal positions in the front quadrants of the sphere to enhance the morphological description of the object. Additional observation points were tested in the experiments, but they did not improve the approximation accuracy (Fig. 1, available as supplementary data at *Bioinformatics* online).

For each observation point the mesh silhouette was calculated by projecting all facets onto the corresponding plane. As a result, binary image encodes projection with 1 and background with 0 is formed for each observation point of spine. To describe obtained projections with decomposition onto Zernike moments, the silhouettes were inscribed into a unit disk. At this stage, the silhouette is normalized by the unit-disk mapping, so no further scaling is needed. Analytically, the decomposition coefficients are derived by projecting the target polar function f(r, θ) onto the basis functions *V*_mn_(*r*, *θ*). Here, *n* ∈ *ℕ*_0_ is the radial order and *m* ∈ ℤ is the azimuthal (angular) order of the Zernike polynomial *V*_mn_. The projecting is described as:


$$a_{mn}=\int_{0}^{1}\int_{0}^{2\pi }f(r,\theta )V_{mn}(r,\theta )d\theta dr$$

Numerically, this integral can be approximated by a finite sum:


$$a_{mn}=\sum \sum_{x^{2}+y^{2}\leq 1}f(x,y)V_{mn}(\sqrt{x^{2}+y^{2}},\arctan\left(\frac{y}{x}\right)),$$

where (*x*, *y*) represents the pixel coordinates of projection image. So the decomposition coefficients are obtained by evaluating this sum for each of the basis functions for the chosen approximation order *M*. The proposed method can be parametrized by the number of observation points and the order of approximation. The optimal parameters were chosen using a grid search technique. The detailed analysis of grid search results is provided in Supplementary Material 1, available as supplementary data at *Bioinformatics* online. The optimal number of observation points was found to be 5, and the optimal approximation order was 10 (Fig. 1B, available as supplementary data at *Bioinformatics* online).

### 2.3 Classification, clustering, visualization, and statistics

For the dataset metric distributions and Hausdorff distance distributions for ellipse and spherical harmonics approximations statistical analysis was conducted with GraphPad Prism software. ML classification approach was performed using SVM, LightGBM and XGboost classification models trained on a dataset manually labeled by 7 experts. Clustering was performed using k-means method. Clustering quality was evaluated using the elbow and silhouette criteria, as well as separability statistics between control and Aβ groups, including the Agresti–Caffo and two-sample chi-squared tests. Dimensionality reduction was performed using UMAP, PCA, and t-SNE, which were also applied for visualization of the morphological distributions. Details of mentioned above methods, all packages and statistical tests are described in Section 1, available as supplementary data at *Bioinformatics* online.

## 3 Results

### 3.1 Spherical harmonics and light field as a new 3D dendritic spine shape descriptors

To achieve a more morphology-sensitive encoding of dendritic spine shapes, a technique using 3D shape descriptors was developed. The first-time proposed descriptors are based on Spherical Harmonics and on Light Field.

**Figure 2:**
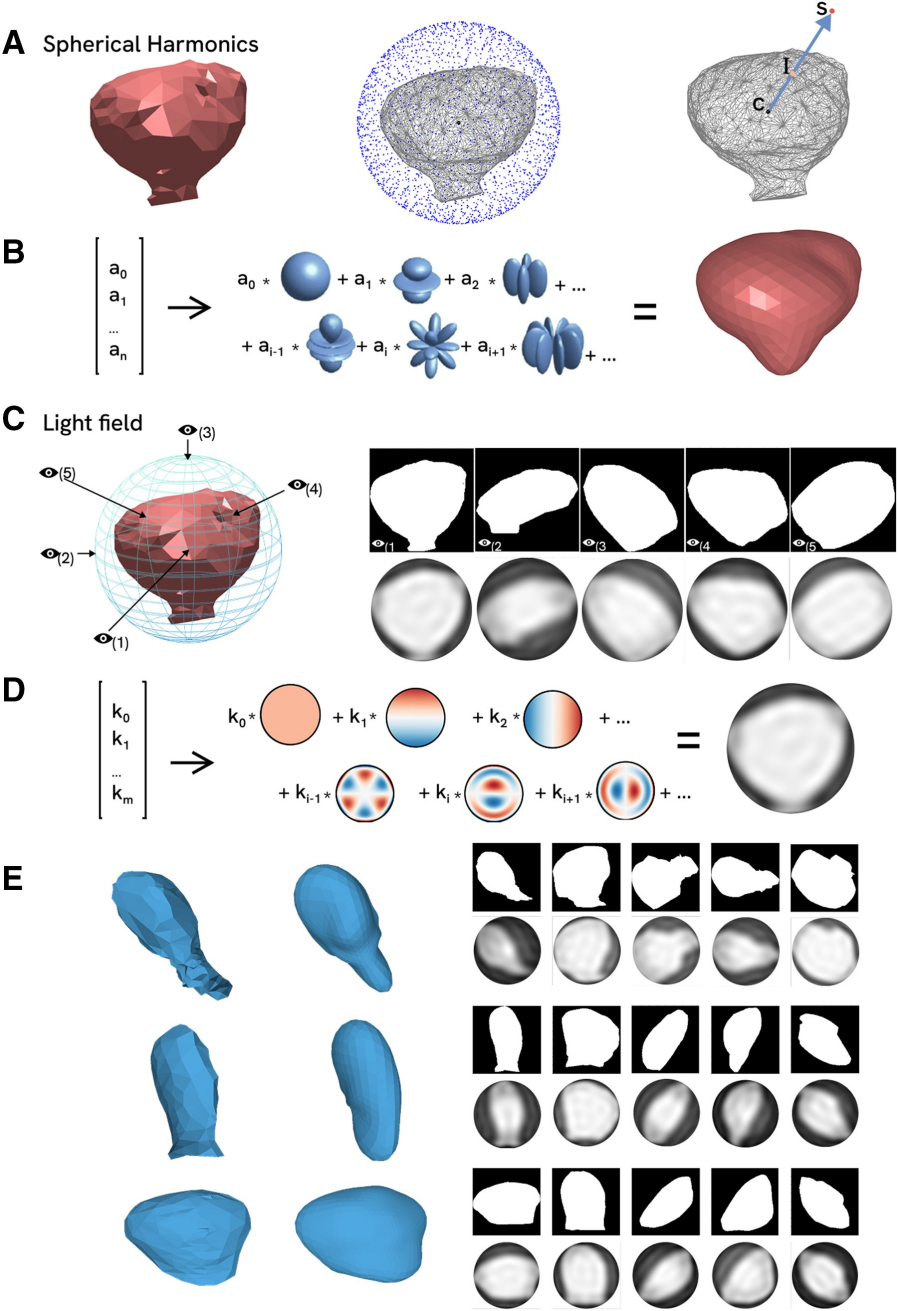
Spherical Harmonics and Light Field as 3D dendritic spine shapes descriptors. (A) Dendritic spine approximation procedure: the polygonal mesh is enclosed by a bounding sphere, points are uniformly sampled on the sphere, and rays are cast from the center (C point) towards these points (S point). The intersections of the rays with the polygonal mesh surface (I point) form a set of approximated points. (B) Sum of basis functions of harmonics results dendritic spine approximation (spheres images are modified from https://doi.org/10.1155/2015/582870, which is distrubited under the Creative Commons Attribution License). (C) Dendritic spine representation: five different viewpoints (marked with eye) are chosen to get the projection of 3D mesh, five 2D binary images for approximation are extracted and spine projections approximations are reconstructed (beneath images). (D) Sum of basis functions of Zernike moments results binary 3D image approximation (Zernike polynomials images are modified from https://commons.wikimedia.org/wiki/File:Zernike_polynomials_with_read-blue_cmap.png, which is distributed under the Creative Commons Attribution-Share Alike 4.0 International). (E) Example of approximation of different types of dendritic spine morphologies with SphHarm on the left and spine projections for LF on the right.

First proposed novel spine shape descriptor is based on the decomposition of spherical harmonic—spherical mathematical functions defined on the surface of a sphere. For spine shape description the mesh preprocessing algorithm was developed. To compute morphology approximation, points on the spine surface were sampled uniformly for each spine mesh (Fig. 2A). As a result, each spine was encoded with a set of coefficients ai, and weighed sum of spherical harmonics represents the approximated spherical surface (Fig. 2B). Correlation analysis showed correlation values between the elements of the vector representation, which indicates that proposed descriptor is informative and non-redundant.

The second shape descriptor we propose to use for dendritic spines morphology analysis is Light Field based onto decomposition on Zernike moments. The special spine mesh preprocessing technique also was developed to use Zernike moments for spines shape encoding (Fig. 2C). As a result, each spine was encoded with five sets of decomposition coefficients ki. The sum of coefficients within one set describes approximated 2D binary image (Fig. 2D). Since the obtained decomposition coefficients are complex numbers, two methods were used to obtain a real-valued representation for further analysis in classification and clustering tasks: extracting the real part of the number and computing the modulus of the complex number. The best results were achieved using the modulus, which was subsequently used in the clustering task.

To compute the encoding of a spine using both proposed descriptors, the spine was oriented in space to ensure comparability of different shapes. The entire dataset was processed, and the corresponding 3D shape metrics were obtained. To visually assess the resulting feature space, methods for reconstructing spine representations from their decomposition coefficients were developed. Several representative spines of different shapes, along with their approximated reconstructed representations, were selected and displayed in the figure (Fig. 2E).

As decomposition-based methods require mesh orientation and scaling, they are invariant to mesh size. Therefore, in some experiments, the scalar classical metric volume, which describes spine size, was added to the feature space. Both classification and clustering accuracy were compared with and without the inclusion of the volume metric. The optimal technique was then selected for specific tasks and methods.

### 3.2 Dendritic spines reconstruction method based on spherical harmonics

Proposed technique of spines surface reconstruction and modulation on base of spherical harmonics decomposition was compared with other method introduced early and relied onto set of oriented ellipses (Luengo-Sanchez *et al.* 2018). Dataset, that we used for decomposition methods comparison, consist of 550 polygonal meshes of the spines and the spines were manually classified onto four classes: mushroom, thin, stubby and filopodia (dataset description is presented Section 2, available as supplementary data at *Bioinformatics* online). We encoded 433 spines from the dataset with both spherical harmonics and oriented ellipses decomposition algorithms, excluding spines for which at least one of decomposition method was not possible, as well as filopodia spines. Overall, for comparison were encoded 213 of mushroom, 113 of stubby and 107 of thin spine types. For each spine approximation accuracy using symmetric Hausdorff distance were measured.

**Figure 3:**
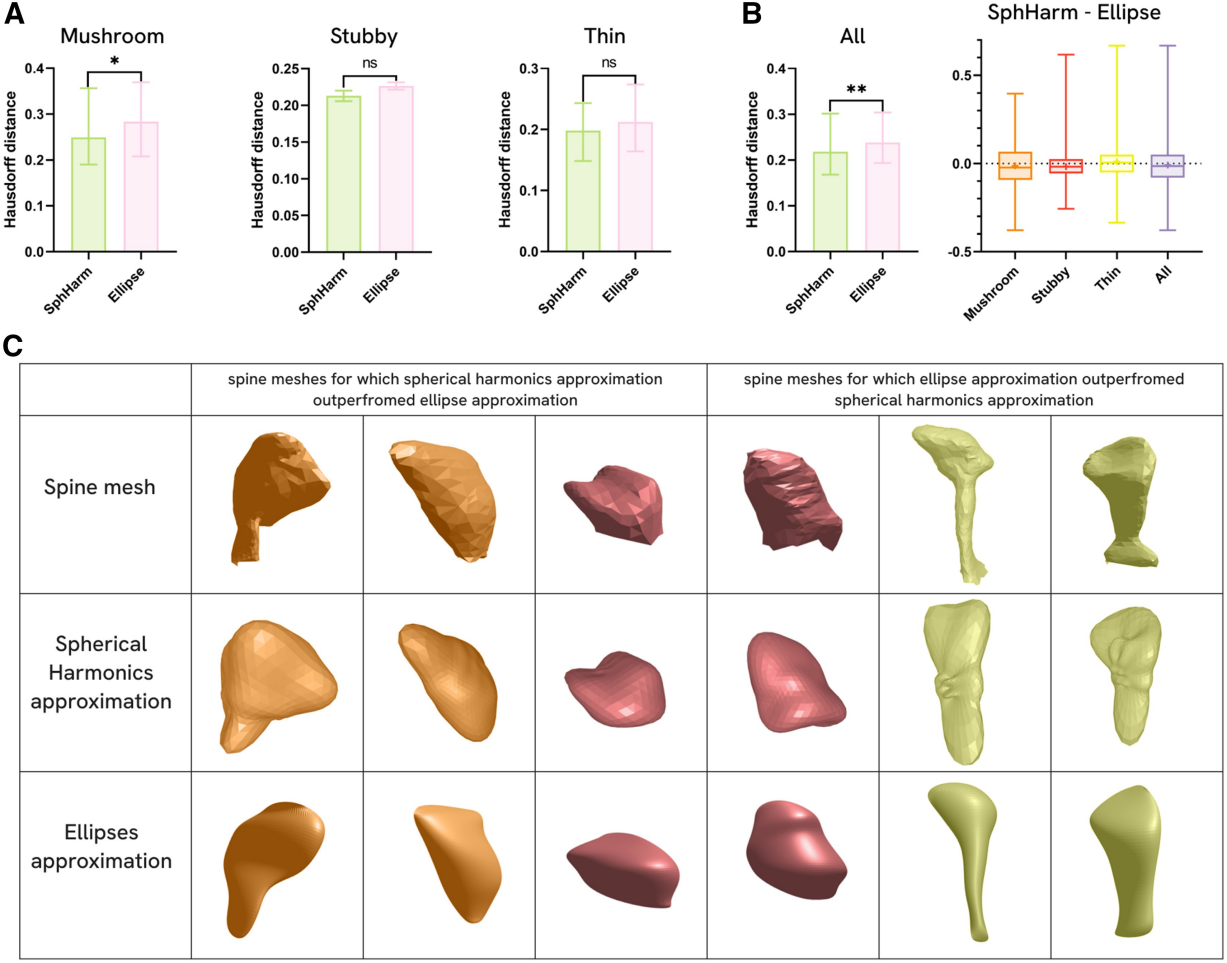
Comparative analysis of dendritic spine shape reconstruction methods. (A) Hausdorff distance distribution comparison of Spherical Harmonics and ellipses approximation techniques within classes and all dataset. For the stubby class the distance is shown as mean±SEM (Welch’s *t*-test). For mushroom, thin and all together spines Hausdorff distance is shown as median with interquartile range [Q1; Q3] (Mann–Whitney test). (B) Distribution of the difference between the Hausdorff distance for Spherical Harmonics approximation and for ellipses approximation across morphological classes. Whiskers are shown as minimal value to maximal, mean is shown as “+.” Negative values indicate dendritic spines types where Spherical Harmonics provide a more accurate shape approximation. (C) Visual comparison of dendritic spine shape reconstruction by spherical harmonics and ellipses approximations for different spine morphologies. The first three columns show spines where Spherical Harmonics provide a more accurate approximation, while the last three columns show cases where ellipsoidal models achieve better accuracy. **P < .01, *P < .05.

Statistical significance of distribution differences was obtained for the class of mushroom spines (0.249 [0.190; 0.356] and 0.284 [0.209; 0.368], *P* = .041) and the combined dataset (0.218 [0.168; 0.300] and 0.238 [0.194; 0.304], *P* = .0013), where Hausdorff distance for Spherical Harmonics approximation is less than for ellipse approximation both (Fig. 3A). No statistical significance of distribution differences was obtained for the stubby class (0.213 ± 0.00716 for spherical harmonics approximation, and 0.226 ± 0.00496 for ellipse approximation, *P* = .123) and the thin class (0.198 [0.151; 0.242] for spherical harmonics approximation, and 0.212 [0.164; 0.272] for ellipse approximation, *P* = .745). To evaluate the difference in approximation accuracy for each individual spine, the difference between the Hausdorff distances of the Spherical Harmonics-based and ellipses-based approximations was computed. On average, spherical harmonics provide a more accurate approximation across all spine groups, although a greater number of outliers correspond to spines for which harmonics yield a less precise approximation compared to ellipses (Fig. 3B). Three representative spines were selected from both the lower and upper outlier regions of the graph to visualize the strengths and limitations of the proposed shape reconstruction and modeling method (Fig. 3C).

### 3.3 3D descriptors are more sensitive for clustering analysis of normal and Alzheimer’s disease modeling dendritic spines in vitro

The collected dataset consists of 550 spines polygonal meshes, 279 of them is control group, 271—Aβ. Detailed dataset description, scalar metrics and manual classification results distribution comparison of control and Aβ groups are explained and visualized in Material 2, available as supplementary data at *Bioinformatics* online and Fig. 2, available as supplementary data at *Bioinformatics* online. Briefly, comparison of scalar metrics between the control and Aβ groups indicates a reduction in spine volume, junction area in Aβ group and differences in other 5 metrics. Manual classification revealed that Aβ group has fewer of the mushroom spine type, that are associated with the long-term memory (Hayashi and Majewska 2005, Bourne and Harris 2007), and more thin and filopodia types. For each class that were manually classified CLDH distributions and shape approximation using proposed descriptors were processed (Fig. 3, available as supplementary data at *Bioinformatics* online).

Furthermore, we applied supervised machine learning to classify the dataset using manually labeled spines and their descriptor values across the four metric spaces: eleven scalar metrics, CLDH values, SphHarm, and LF. As supervised classification remains a widely used tool in studies of spine morphology and plasticity, we also examined how well each descriptor family supports this analysis. The accuracy of machine classification indicates the separability of the metric space. For the dataset, machine classification based on the SphHarm and LF demonstrates that their respective spaces exhibit separability comparable to the scalar metrics space and superior to the CLDH values space (see Material 3, available as supplementary data at *Bioinformatics* online and Table 1, available as supplementary data at *Bioinformatics* online).

**Table 1:** Best performance of each descriptor in clustering.^a^

Dimension	Number of clusters	How many clusters are statistically different (*P*-value of Agresti-Caffo test > .05)	What are *P*-values of Agresti-Caffo for this clusters	Chi-square *P*-value
Classic, tSNE	7	2	0.0370; 0.000802	0.00390
Chord, PCA/no dim. reduction	7	1	0.0373/0.0206	0.282/0.201
SphHarm, PCA	5	3	0.0473; 0.00161; 0.0473	0.00262
**SphHarm + Volume, UMAP**	**6**	**4**	**0.0311; 0.0203; 0.00705; 0.00607**	**0.000915**
LF real values, PCA	6	3	0.0186; 0.0363; 0.0339	0.0114
LF real values + Volume, no dim. reduction	4	3	0.0471; 0.00704; 0.0000913	0.000109
**LF modulus, no dim. reduction**	**5**	**4**	**0.0432; 0.0446; 0.000116; 0.000811**	**0.00000331**
LF modulus + Volume, UMAP	5	2	0.00647; 0.000147	0.000294

a The best results for separability are highlighted in bold.

**Figure 4:**
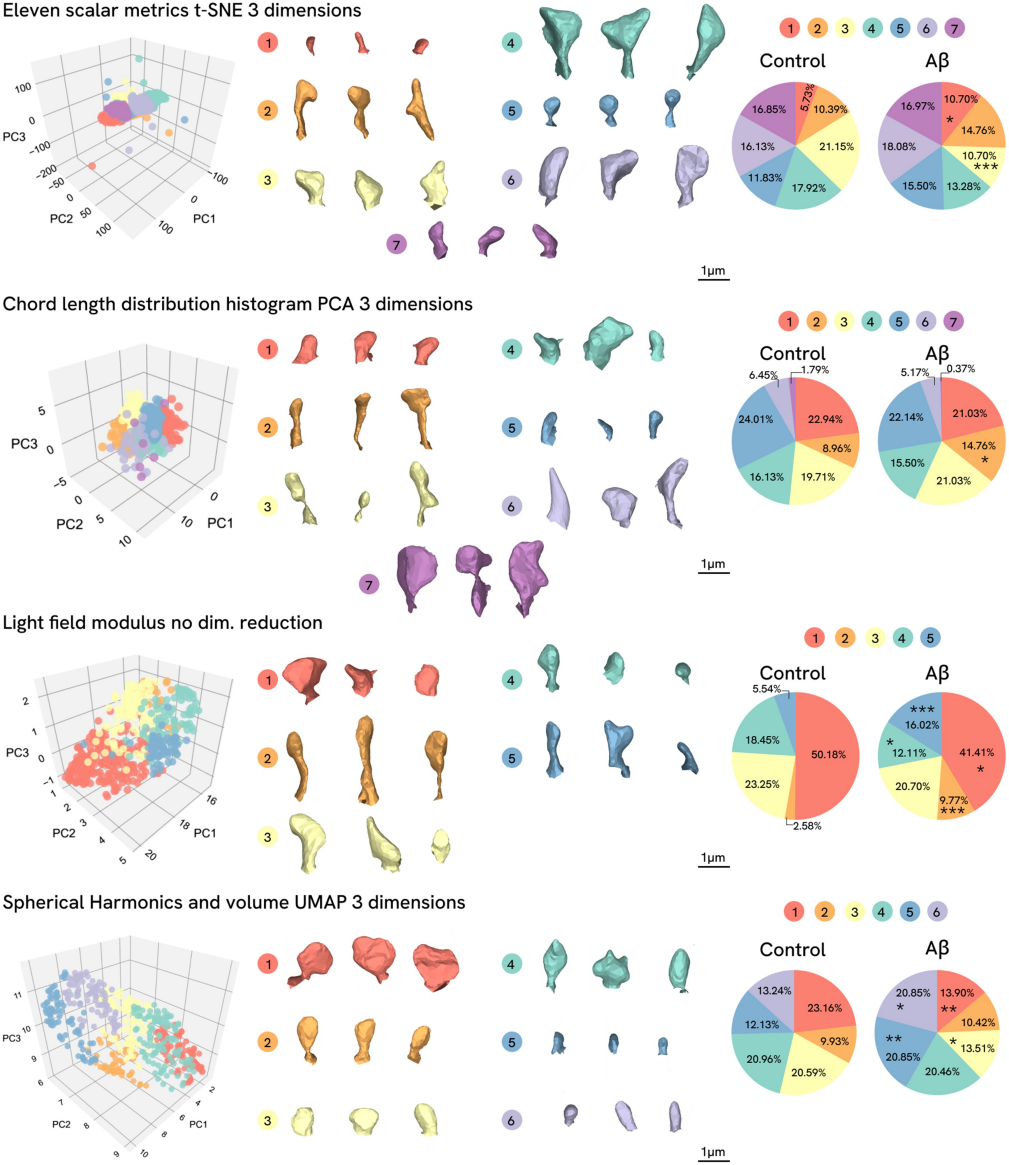
Spine clustering results for different descriptors using k-means clustering algorithm. On the left side—spines clustering maps with every metric in 3D coordinates using reduction method indicated in the figure, for clustering without dimensionality reduction, the 3D coordinates are represented using the UMAP reduction method. 3D plots codes are presented in the supplementary data. In the middle—representative spines for each cluster. On the right side—pie chart representation of the cluster distribution in the control and experimental group. For the control/Aβ comparison Agresti-Caffo independence test used. ***P < .001, **P < .01, *P < .05.

Spines were clustered based on the values of all four mentioned descriptors (set of 11 scalar metrics, CLDH, SphHarm and LF) with k-means algorithm, for the SphHarm and LF metrics, reduced datasets were used for clustering since it was not possible to calculate them for all polygonal meshes (531 and 527 spines, respectively). For each descriptor clustering without dimensional reduction and with reduction to three dimensions using three methods [Principal Component Analysis (PCA), t-distributed Stochastic Neighbor Embedding (t-SNE) and Uniform Manifold Approximation and Projection (UMAP)] was performed. Optimal numbers of clusters were chosen by the elbow score and silhouette both, metrics scores are collected in one graph for each result (Figs 4 and 5, available as supplementary data at *Bioinformatics* online). All clustering results and description of them are presented in Material 4, available as supplementary data at *Bioinformatics* online and Table 2, available as supplementary data at *Bioinformatics* online. For each descriptor clustering result were chosen in particular space according to best performance (Table 1), meaning the best control and Aβ spines group separability (using independence Agresti-Caffo test between clusters distribution in control/Aβ and chi-squared test between control/Aβ distribution in clusters). For CLDH descriptor, PCA 3D and without dimensional reduction results are statistically identical, they both presented in Table 1. Same method was used to choose the best clustering result in SphHarm and LF descriptors clustering (which are presented on Fig. 4, other clustering results using proposed descriptors are presented on Figs S6 and S7, available as supplementary data at *Bioinformatics* online). For clustering using scalar metrics—t-SNE 3D reduction was selected, for SphHarm with volume—UMAP 3D and for Light field modulus there was no dimensional reduction before clustering (Fig. 4). For CLDH clustering with two statistically identical best results, Fig. 4 presents PCA 3D reduction result. Thus, the best clustering results of each descriptor are compared between them. Representative spines of each clustering result was chosen to analyze morphologies specificity. Different dimensionality reduction methods were found to perform optimally for different shape descriptors in our dataset, suggesting intrinsic structural differences between the descriptors. To explore these effects, we analyzed several dimensionality reduction techniques, and the results are provided in Section 4, available as supplementary data at *Bioinformatics* online.

Clusters distribution in control/Aβ groups were compared between each other for all descriptors using independence Agresti-Caffo test. For clustering using eleven scalar metrics out of 7 clusters, only 2 are significantly different distributed: the proportion of spines in the first cluster increased from 5.73% in the control group to 10.70% in Aβ group of spines (*P* = .037), while the proportion of spines in the third cluster decreased from 21.15% in the control group to 10.70% in Aβ group (*P* = .0008). For CLDH metric clustering only one cluster is predominantly composed of such spines. For light field modulus 4 clusters out of 5 are differently distributed: there are increasing from 2.58% in the control group to 9.77% in Aβ group in the 2 cluster (*P* = .0008), as well as in 5 cluster from 5.54% in the control group to 16.02% in the Aβ group (*P* = .0001). In the first and fourth clusters, there is a decrease from the control group to the Aβ group: from 50.18% to 41.41% in the first cluster (*P* = .043) and from 18.45% to 12.11% in the fourth cluster (*P* = .045). With spherical harmonics and volume clustering there are 4 out of 6 clusters that have differences in distributions: they are increasing from 12.13% in the control group to 20.85% in Aβ group in the 5 cluster (*P* = .007), as well as in 6 cluster from 13.24% in the control group to 20.85% in the Aβ group (*P* = .020). In the first and third clusters there are decreasing from control group to Aβ: from 23.16% to 13.90% in the first cluster (*P* = .006) and from 20.59% to 13.51% in the third (*P* = .031). Thus, for 3D descriptors, separability in a greater number of clusters between control and Aβ was observed than for the previously proposed metric spaces. We hypothesize that the better separation of normal and pathological spines in the clusters indicates that the new descriptors are more sensitive to spine shape alterations. This heightened sensitivity is particularly useful for experimental tasks aimed at assessing the impact of substances or stimuli on dendritic spine morphology.

To validate the obtained results, clustering was also performed on the control group alone. The results are presented in Supplementary Material 4, available as supplementary data at *Bioinformatics* online (Fig. 8, available as supplementary data at *Bioinformatics* online and Table 3, available as supplementary data at *Bioinformatics* online), and show that with probability exceeding 90%, the separation of control/Aβ spine in clusters in the main clustering performances is non-random.

Since reduced datasets were used for clustering with the new descriptors compared to the set of scalar metrics and CLDH, clustering based on scalar metrics and CLDH was also performed on the reduced dataset used for the light field descriptor clustering (527 spines). The results of the Agresti-Caffo and Chi-square tests did not show differences from the results of these tests of the clustering on the dataset of 550 spines (Table 4, available as supplementary data at *Bioinformatics* online), which indicates the validity of comparing the main results with each other.

## 4 Discussion

In this study, we introduced novel methods for analyzing spine morphology and demonstrated their enhanced effectiveness in distinguishing between normal and Aβ spines in clustering results, surpassing traditional classification methods. We used various shape descriptors to characterize spine morphology, including newly developed 3D methods that had not previously been applied to dendritic spines. These methods showed significant effectiveness in both classification and clustering tasks. These findings highlight the potential of advanced morphological analysis to provide deeper insights into the structural changes associated with neurological conditions.

The novel spine shape descriptors developed in this study possess an inherently complex structure, enabling a more precise encoding of morphological features. Usage of descriptor provide us a technique of identifying the central most probable morphology within spines grouping such as clustering and classification. Furthermore, SphHarm and LF in comparison with existing methods of morphology analysis extend results examination with analysis of mean representation of group. Scalar metrics could be visualized and evaluated with boxplots, a more detailed and complex metric CLDH can be represented as an averaged histogram. LF descriptor allows for the generation of an averaged representation of the object’s 2D projections.

Finally, SphHarm metric enables the reconstruction of the full averaged morphology, approximating it as a surface. This capability allows for the incorporation of local morphological characteristics and facilitates the reconstruction, modeling, and statistical generation of dendritic spines as surfaces in spherical coordinates, which can subsequently be converted into mesh representations. The results of comparing the approximation potential of spherical harmonics with the only currently available shape reconstruction method, method of approximation with ellipses (Luengo-Sanchez *et al.* 2018), showed that, on average, spherical harmonics achieves higher accuracy in surface approximation. While the proposed method encounters challenges in approximating the shape of thin spines due to its intrinsic structure and the limitations of spherical coordinate representation, it provides a more precise reconstruction of local features, such as small indentations in the spine’s head (Fig. 3) and preserves sufficient discriminative power to identify thin spines as a distinct morphological group. LF descriptor can, in principle, reconstruct the original 3D shape from its approximated projections. However, since it lacks an explicit procedure for mapping 2D projections back into 3D space, this process requires the development of a dedicated reconstruction methodology to ensure accurate and consistent recovery of the underlying geometry.

Although scalar metrics remain highly accurate for classifying dendritic spines into mushroom, thin, and stubby types, the proposed descriptor based on Spherical Harmonics demonstrates comparable classification accuracy (Table 1, available as supplementary data at *Bioinformatics* online).

Clustering approach seems to us neater tool since spine morphology lately is considered as the continuum of shapes (Ofer *et al.* 2022, Schünemann *et al.* 2025). Previously, several studies used clustering for spines morphology analysis. The research (Pchitskaya and Bezprozvanny 2020) proposes the application of cluster analysis methods to morphological descriptors to derive an optimal partitioning of dendritic spines. Similarly, Gemin *et al.* (2021) applies a continuum-based approach, where the analysis of morphological feature distributions and the investigation of synaptic plasticity are conducted by comparing distributions of morphological characteristics among different dendritic spine populations, rather than relying on the traditional classification into three or four historically established groups. Studies investigating optimal clustering approaches for dendritic spines based on morphological descriptors typically identify 7 to 10 clusters, depending on the applied clustering method (Bokota *et al.* 2016). A recent study examining dendritic responses to olfactory learning further emphasized the advantages of clustering over traditional classification in dendritic spine analysis. In that study, the optimal number of clusters was determined to be five, with clustering performed based on scalar morphological features (Ferreira *et al.* 2024). Clustering was used to group dendritic spines based on their head size, allowing for the identification of natural patterns in size variations and responses to stimulation (Henry *et al.* 2017).

Analysis of metric spaces that was formed by computing classical metrics of dendritic spines morphology and CLDH metric proposed in SpineTool shows that there is separation of the control and Aβ spines of the dataset we used. The most significant classical scalar metrics differing between control and Aβ spines are open angle, junction area, length to volume ratio and length to area ratio (*P* < .0001). The metric values indicate that dendritic spines in amyloid toxicity tend to have an elongated morphology, characterized by a high length-to-volume ratio and a low open-angle metric. Manual classification approach showed us that the proportion of the filopodia spines, that are usually not functional and capable of transforming into other types of spines (Berry and Nedivi 2017, Mao *et al.* 2018) is increasing in neurons with in in vitro AD model. This is consistent with previous observations in the studied model of amyloid toxicity (Popugaeva *et al.* 2015, Pchitskaya *et al.* 2017, Rakovskaya *et al.* 2025), but gives deeper insight in the spine shape changes. Difference of average CLDH for Aβ type and control spines shows that Aβ type spines have a greater probability to be shorter than a third of their length while control spines are more inclined to have multiple chords larger than half the length (Fig. 1B, available as supplementary data at *Bioinformatics* online).

Clustering the spines using new descriptors we see several distinctive forms between control and Aβ spines that are not really similar to traditionally used classes (Fig. 4). Elongated large objects and large non-classical shaped objects form separate clusters in Light Field modulus metric space—clusters 2 and 5 respectively. These clusters have significantly different proportions spines groups, and Aβ is prevalent there. Meanwhile, more spines of control group occurred in 1 and 4 clusters, where 1 cluster represents spines that are similar to mushroom subtype (big head surface), and spines in 4 cluster are similar both to thin and stubby subtypes. In combined feature space of Spherical Harmonics and volume small spines are divided into two clusters, cluster 5 and cluster 6, spines in sixth cluster have similarities with stubby subtype, while 5 cluster’s spines look more shortened. In these clusters, Aβ spines are more prevalent. Moreover, in this clustering control spines predominate in first and third clusters, and spines in them have bigger size of the head and overall. Such results are consistent with past assertions, that during Alzheimer’s disease dendritic spines are shrinked and eliminated in hippocampus (Boros *et al.* 2017) and give more points to the assumption that dendritic spine degeneration and elimination passes through a stage of transformation into stubby subtype spines (Flores *et al.* 2025). K-means clustering approach showed us that scalar metric clustering may give more shape morphologies, than clustering in SphHarm and LF spaces (7 clusters with set of scalar and CLDH and 5–6 with new descriptors), but less differences between control/Aβ groups distribution in clusters. In CLDH metric space a statistical distinction between groups only in one cluster, which can be attributed to the size-insensitivity and symmetry of the chord distribution regard to spine form. Thus, we hypothesize that the better the separability of normal and pathological spines in clusters, the more sensitive the proposed descriptor, and the more accurately the effects of potential therapeutic agents can be assessed. For this reason, the proposed descriptors can be used to evaluate changes in dendritic spine morphology in preclinical studies. They are supposed to be particularly useful for studying dendritic spine changes in response to various physiological situations where changes are subtler than in pathological conditions. These descriptors can detect changes that are inaccessible with traditional classification methods and where direct morphometric measurement of all spines cannot provide insights into specific shape alterations.

As future direction of our work we define improvement of spherical harmonics-based descriptor to encode non-spherical function and resolve limits for thin spines description. We also see the potential of spherical harmonics in generation of synthetic datasets. These datasets could be used to improve machine learning methods accuracy and enhance set of methods available in the field of spines analysis. Since clustering analysis has demonstrated a higher accuracy in distinguishing control and Aβ groups morphologies, we plan to continue analysis of other clustering methods and other datasets from various brain regions and species. As part of future work, we plan to systematically investigate how inaccuracies in shape description propagate through the analysis pipeline and influence the outcomes of morphological clustering and interpretation. Considering the differences in the distribution of spine classes among dendrites (Theobald *et al.* 2025), one of the research directions could be the refinement of those findings using new descriptors and cluster approach. The future direction is also to expand the conclusions regarding the resulting morphological groups by linking them to the spine function and the effect on synaptic plasticity in various fundamental neuroscience research. As novel methods were developed on the base of SpineTool module, an additional direction of work will involve integrating the methods into the software.

## Supplementary Material

btag025_Supplementary_Data

## Data Availability

SpineTool software used for experiments is public and available at https://github.com/Biomed-imaging-lab/SpineTool with the DOI: 10.5281/zenodo.17359066. SpineTool software tutorial can be found at https://doi.org/10.1002/cpz1.70061. Descriptors codebase is available at https://github.com/Biomed-imaging-lab/Spine-Shape-Descriptors with the DOI: 10.5281/zenodo.17302859. For any other information or requests for data that is not listed above or in the supplementary materials, please contact Daria Smirnova and the corresponding author Ekaterina Pchitskaya.

## References

[btag025-B1] Arellano JI , Benavides-Piccione R, Defelipe J et al Ultrastructure of dendritic spines: correlation between synaptic and spine morphologies. Front Neurosci 2007;1:131–43. 18982124 10.3389/neuro.01.1.1.010.2007 PMC2518053

[btag025-B2] Ashokan A , Lim JWH, Hang N et al Complex housing causes a robust increase in dendritic complexity and spine density of medial prefrontal cortical neurons. Sci Rep 2018;8:7308. 29743496 10.1038/s41598-018-25399-4 PMC5943332

[btag025-B3] Bailey CH , Kandel ER, Harris KM. Structural components of synaptic plasticity and memory consolidation. Cold Spring Harb Perspect Biol 2015;7:a021758. 26134321 10.1101/cshperspect.a021758 PMC4484970

[btag025-B4] Barrientos C , Knowland D, Wu MMJ et al Cocaine-induced structural plasticity in input regions to distinct cell types in nucleus accumbens. Biol Psychiatry 2018;84:893–904. 29921416 10.1016/j.biopsych.2018.04.019 PMC8169057

[btag025-B5] Berry KP , Nedivi E. Spine dynamics: are they all the same? Neuron 2017;96:43–55. 28957675 10.1016/j.neuron.2017.08.008 PMC5661952

[btag025-B6] Bhatt DH , Zhang S, Gan WB. Dendritic spine dynamics. Annu Rev Physiol 2009;71:261–82. 19575680 10.1146/annurev.physiol.010908.163140

[btag025-B7] Bokota G , Magnowska M, Kuśmierczyk T et al Computational approach to dendritic spine taxonomy and shape transition analysis. Front Comput Neurosci 2016;10:140. 28066226 10.3389/fncom.2016.00140 PMC5180374

[btag025-B8] Boros BD , Greathouse KM, Gentry EG et al Dendritic spines provide cognitive resilience against Alzheimer’s disease. Ann Neurol 2017;82:602–14. 28921611 10.1002/ana.25049 PMC5744899

[btag025-B9] Bourne J , Harris KM. Do thin spines learn to be mushroom spines that remember? Curr Opin Neurobiol 2007;17:381–6. 17498943 10.1016/j.conb.2007.04.009

[btag025-B10] Bourne JN , Harris KM. Balancing structure and function at hippocampal dendritic spines. Annu Rev Neurosci 2008;31:47–67. 18284372 10.1146/annurev.neuro.31.060407.125646 PMC2561948

[btag025-B11] Chidambaram SB , Rathipriya AG, Bolla SR et al Dendritic spines: revisiting the physiological role. Prog Neuropsychopharmacol Biol Psychiatry 2019;92:161–93. 30654089 10.1016/j.pnpbp.2019.01.005

[btag025-B12] Dimov IT , Penzov AA, Stoilova SS. Parallel Monte Carlo sampling scheme for sphere and hemisphere. In: Boyanov T, Dimova S, Georgiev K et al (eds) Lecture Notes in Computer Science (Including Subseries Lecture Notes in Artificial Intelligence and Lecture Notes in Bioinformatics). Berlin, Heidelberg: Springer Verlag, 2007, 148–55.

[btag025-B13] Ferreira A , Constantinescu V-S, Malvaut S et al Distinct forms of structural plasticity of adult-born interneuron spines in the mouse olfactory bulb induced by different odor learning paradigms. Commun Biol 2024;7:420. 38582915 10.1038/s42003-024-06115-7 PMC10998910

[btag025-B14] Flores G , Aguilar-Hernández L, García-Dolores F et al Dendritic spine degeneration: a primary mechanism in the aging process. Neural Regen Res 2025;20:1696–8. 39104099 10.4103/NRR.NRR-D-24-00311 PMC11688554

[btag025-B15] Gemin O , Serna P, Zamith J et al Unique properties of dually innervated dendritic spines in pyramidal neurons of the somatosensory cortex uncovered by 3d correlative light and electron microscopy. PLoS Biol 2021;19:e3001375. 34428203 10.1371/journal.pbio.3001375 PMC8415616

[btag025-B16] Ghani MU, Erdil E, Kanık SD et al Dendritic spine shape analysis: a clustering perspective. In: Hua G, Jégou H (eds), Lecture Notes in Computer Science (Including Subseries Lecture Notes in Artificial Intelligence and Lecture Notes in Bioinformatics), Vol. 9913. LNCS, Cham, Switzerland: Springer, 2016, 256–73.

[btag025-B17] Hayashi Y , Majewska AK. Dendritic spine geometry: functional implication and regulation. Neuron 2005;46:529–32. 15944122 10.1016/j.neuron.2005.05.006

[btag025-B18] Henry FE , Hockeimer W, Chen A et al Mechanistic target of rapamycin is necessary for changes in dendritic spine morphology associated with long-term potentiation. Mol Brain 2017;10:50. 29084578 10.1186/s13041-017-0330-y PMC5663037

[btag025-B19] Kashiwagi Y , Higashi T, Obashi K et al Computational geometry analysis of dendritic spines by structured illumination microscopy. Nat Commun 2019;10:1285. 30894537 10.1038/s41467-019-09337-0 PMC6427002

[btag025-B20] Loewenstein Y , Kuras A, Rumpel S. Multiplicative dynamics underlie the emergence of the log-normal distribution of spine sizes in the neocortex in vivo. J Neurosci 2011;31:9481–8. 21715613 10.1523/JNEUROSCI.6130-10.2011 PMC6623170

[btag025-B21] Luengo-Sanchez S , Fernaud-Espinosa I, Bielza C et al 3D morphology-based clustering and simulation of human pyramidal cell dendritic spines. PLoS Comput Biol 2018;14:e1006221. 29897896 10.1371/journal.pcbi.1006221 PMC6060563

[btag025-B22] Mao Y-T , Zhu JX, Hanamura K et al Filopodia conduct target selection in cortical neurons using differences in signal kinetics of a single kinase. Neuron 2018;98:767–82.e768. 29731254 10.1016/j.neuron.2018.04.011 PMC5987257

[btag025-B23] Medyukhina A , Blickensdorf M, Cseresnyés Z et al Dynamic spherical harmonics approach for shape classification of migrating cells. Sci Rep 2020;10:6072. 32269257 10.1038/s41598-020-62997-7 PMC7142146

[btag025-B24] Nakahata Y , Yasuda R. Plasticity of spine structure: local signaling, translation and cytoskeletal reorganization. Front Synaptic Neurosci 2018;10:29. 30210329 10.3389/fnsyn.2018.00029 PMC6123351

[btag025-B25] Ofer N , Benavides-Piccione R, DeFelipe J et al Structural analysis of human and mouse dendritic spines reveals a morphological continuum and differences across ages and species. eNeuro 2022;9:ENEURO.0039–22.2022. 10.1523/ENEURO.0039-22.2022 PMC9186112 35610025

[btag025-B26] Ofer N , Berger DR, Kasthuri N et al Ultrastructural analysis of dendritic spine necks reveals a continuum of spine morphologies. Dev Neurobiol 2021;81:746–57. 33977655 10.1002/dneu.22829 PMC8852350

[btag025-B27] Pchitskaya E , Bezprozvanny I. Dendritic spines shape analysis—classification or clusterization? Perspective. Front Synaptic Neurosci 2020;12:31. 33117142 10.3389/fnsyn.2020.00031 PMC7561369

[btag025-B28] Pchitskaya E , Kraskovskaya N, Chernyuk D et al Stim2-eb3 association and morphology of dendritic spines in hippocampal neurons. Sci Rep 2017;7:17625. 29247211 10.1038/s41598-017-17762-8 PMC5732248

[btag025-B29] Pchitskaya E , Vasiliev P, Smirnova D et al Spinetool is an open-source software for analysis of morphology of dendritic spines. Sci Rep 2023;13:10561. 37386071 10.1038/s41598-023-37406-4 PMC10310755

[btag025-B30] Penzes P , Cahill ME, Jones KA et al Dendritic spine pathology in neuropsychiatric disorders. Nat Neurosci 2011;14:285–93. 21346746 10.1038/nn.2741 PMC3530413

[btag025-B31] Popugaeva E , Pchitskaya E, Speshilova A et al Stim2 protects hippocampal mushroom spines from amyloid synaptotoxicity. Mol Neurodegener 2015;10:37. 26275606 10.1186/s13024-015-0034-7 PMC4536802

[btag025-B32] Rakovskaya A , Volkova E, Bezprozvanny I et al Hippocampal dendritic spines store-operated calcium entry and endoplasmic reticulum content is dynamic microtubule dependent. Sci Rep 2025;15:1314. 39779788 10.1038/s41598-024-85024-5 PMC11711194

[btag025-B33] Rodriguez A , Ehlenberger DB, Dickstein DL et al Automated three-dimensional detection and shape classification of dendritic spines from fluorescence microscopy images. PLoS One 2008;3:e1997. 18431482 10.1371/journal.pone.0001997 PMC2292261

[btag025-B34] Saraceno GE , Castilla R, Barreto GE et al Hippocampal dendritic spines modifications induced by perinatal asphyxia. Neural Plast 2012;2012:873532. 22645692 10.1155/2012/873532 PMC3356716

[btag025-B35] Schünemann KD , Hattingh RM, Verhoog MB et al Comprehensive analysis of human dendritic spine morphology and density. J Neurophysiol 2025;133:1086–102. 40013734 10.1152/jn.00622.2024

[btag025-B36] Sharma S , Khanna P. Computer-aided diagnosis of malignant mammograms using zernike moments and SVM. J Digit Imaging 2015;28:77–90. 25005867 10.1007/s10278-014-9719-7 PMC4305050

[btag025-B37] Smith KR , Kopeikina KJ, Fawcett-Patel JM et al Psychiatric risk factor ank3/ankyrin-g nanodomains regulate the structure and function of glutamatergic synapses. Neuron 2014;84:399–415. 25374361 10.1016/j.neuron.2014.10.010 PMC4223651

[btag025-B38] Steffens H , Mott AC, Li S et al Stable but not rigid: chronic in vivo sted nanoscopy reveals extensive remodeling of spines, indicating multiple drivers of plasticity. Sci Adv 2021;7:eabf2806. 34108204 10.1126/sciadv.abf2806 PMC8189587

[btag025-B39] Stein IS , Zito K. Dendritic spine elimination: molecular mechanisms and implications. Neuroscientist 2019;25:27–47. 29716431 10.1177/1073858418769644 PMC6167191

[btag025-B40] Styner M , Oguz I, Xu S et al Framework for the statistical shape analysis of brain structures using spharm-pdm. Insight J 2006:242–50. 21941375 PMC3062073

[btag025-B41] Tahmasbi A , Saki F, Shokouhi SB. Classification of benign and malignant masses based on zernike moments. Comput Biol Med 2011;41:726–35. 21722886 10.1016/j.compbiomed.2011.06.009

[btag025-B42] Theobald CC , Lotfinia A, Knobloch JA et al Distribution of spine classes shows intra-neuronal dendritic heterogeneity in mouse cortex. Neurophotonics 2025;12:015001. 39712647 10.1117/1.NPh.12.1.015001 PMC11657875

[btag025-B43] Ustinova A , Volkova E, Rakovskaya A et al Generate and analyze three-dimensional dendritic spine morphology datasets with spinetool software. Curr Protoc 2024;4:e70061. 39641661 10.1002/cpz1.70061

[btag025-B44] Yuste R , Bonhoeffer T. Morphological changes in dendritic spines associated with long-term synaptic plasticity. Annu Rev Neurosci 2001;24:1071–89. 11520928 10.1146/annurev.neuro.24.1.1071

