# Supplementary material for: 3D dendritic spines shape descriptors for efficient classification and morphology analysis in control and Alzheimer’s disease modeling neurons
Source: Bioinformatics. 2026 Jan 20;42(2):btag025. doi: 10.1093/bioinformatics/btag025 (PMC12891915; doi:10.1093/bioinformatics/btag025)
Supplement: btag025_Supplementary_Data [file btag025_supplementary_data.zip › Supplementary_material.pdf]

Supplementary material

1. Materials and methods 2
2. Dataset description 7
3. Manual classification results shape approximation and ML classification 10
4. Clustering results information 12
5. Reference 22

1. Materials and methods

1.1 Primary hippocampal cultures

All animal procedures were approved by the Bioethics Committee of the Peter the Great St. Petersburg Polytechnic University at St. Petersburg, Russia and followed the principles of European convention (Strasbourg, 1986) and the Declaration of International medical association about humane treatment of animals (Helsinki, 1996). Primary hippocampal neuronal cultures of dissociated hippocampal cells were prepared from newborn Albino inbred mice (FVB/NJ), which were obtained from the Jackson Laboratory (Jackson Laboratory, Bar Harbor, ME, USA, strain #001800) and maintained in culture as described previously (Popugaeva, et al., 2015). Briefly, the hippocampus from postnatal day 0–1 was incubated with papain solution for 30 min at 37 °C (Worthington Biochemical Corp., Lakewood, NJ, USA, #3176) then dissociated with a solution of deoxyribonuclease I (5 mg/mL; Macherey Nagel GMBH, Germany, #R1542S). The neurons were placed in a 24-well plate on 12 mm glass cover glasses pre-coated with 1% poly-D-lysine (Sigma-Aldrich, St Louis, MO, USA, #p-7886). Cells were grown in 1 mL Neurobasal-A (Thermo Fisher Scientific, Waltham, MA, USA, #10888022) supplemented with 2% B27 (Thermo Fisher Scientific, Waltham, MA, USA, #17504044), 1% heat-inactivated fetal bovine serum (FBS, Thermo Fisher Scientific, Waltham, MA, USA, #10500064), and 0.5 mM L-glutamine (Thermo Fisher Scientific, Waltham, MA, USA, #25030024), and were maintained at 37 °C in a 5% CO2 incubator in a 24-well glass plate. Transfection was performed according to (Sun, et al., 2014) with a calcium transfection kit purchased from Clontech (Takara Bio, Kusatsu, Japan, #631312) with pLV-eGFP plasmid that was purchased from the Addgene plasmid database (Addgene, USA, #36083). Preparation of the oligomeric beta-amyloid was described in (Popugaeva, et al., 2015). Cells were incubated with Aβ at a terminal concentration 1 µM for 72 h before being fixed. At DIV16-17 hippocampal primary neurons were fixed in 4% paraformaldehyde (PFA) (Sigma-Aldrich, #158127) and 4% sucrose in PBS, pH 7.3, solution for further analysis.

1.2 Confocal microscopy and image preprocessing

For assessment of the dendritic spine morphology, a Z-stack of the optical section was captured with a confocal microscope (Leica TCS SP8). For dendritic analysis, 2048 × 2048-pixel images with a 0.022 µm/pixel resolution were captured with Z interval of 0.1 µm using a 100× objective lens (NA=1,4, UPlanSApo; Olympus, Tokyo, Japan). The images were pre-processed with ImageJ's built-in "Median" filter to remove noise. In the ImageJ program using the DeconvolutionLab plugin (Sage, et al., 2017) deconvolution was performed for each series using the Richardson-Lucy Total Variation method (regularization parameter 0.1; number of iterations 10).

1.3 Spines segmentation and analysis in SpineTool

Segmentation

To extract dendrites and dendritic spines meshes from microscopy images segmentation methods provided by SpineTool (Pchitskaya, et al., 2023) was used. Each image was processed with analysis pipeline: image binarization, connectivity component choosing, dendrite surface reconstruction and

spines segmentation. SpineTool segmentation requires to set following parameters: base threshold of binarization, local threshold block size, threshold weight, sensitivity and correction of spines segmentation. On each stage of pipelines corresponding parameters was chosen by expert after visual and expert examination.

Dataset metrics evaluation

Extracted spines were manually classified by 7 experts in neuroscience in SpineTool software. The class to which experts more often attributed a specific spine was eventually assigned to this spine. Consensus for each spine were found using function in the SpineTool software. The following spine metrics was extracted with SpineTool: chord length distribution histogram and 11 classical scalar metrics – length, volume, surface area, convex hull volume, convex hull ratio, average distance, coefficient of variance in distance, open angle, length to volume ratio, length to surface area ratio, foot area.

Morphology spectrum analysis methods

For the visualization of metric spaces and clustering results, as well as for improving clustering accuracy in certain metric spaces, several dimensionality reduction methods were employed, including Principal Component Analysis (PCA), t-SNE, and UMAP. All three methods construct a lower-dimensional space that represents the original data while minimizing the loss of mutual distances and separability. These methods rely on the spatial arrangement of data points in the original high-dimensional space.

Clustering is an unsupervised learning task. The k-means algorithm has demonstrated the best performance in both previous experiments on dendritic spine grouping and current studies. The k-means method partitions data into k clusters by iteratively assigning each data point to the nearest cluster centroid and updating centroids based on the mean position of assigned points. This process continues until convergence, minimizing intra-cluster variance (Likas, et al., 2003).

K-means is parametrized by the desired number of clusters. To determine the optimal number of clusters, various clustering quality metrics for dendritic spine grouping are computed.

<u>The elbow method</u> evaluates the sum of squared distances between data points and their assigned cluster centroids as a function of the number of clusters. The total variance typically decreases as the number of clusters increases, but after a certain point, the rate of decrease slows down. The optimal number of clusters is determined at the "elbow" of the curve, where adding more clusters provides diminishing improvements. The inflection point is identified using maximum curvature, which can be obtained by analyzing the second derivative of the metric.

<u>The silhouette method</u> quantifies the cohesion and separation of clusters by computing the silhouette coefficient for each data point. The silhouette coefficient is defined as:

$$S(i) = \frac{|b(i) - a(i)|}{\max\big(a(i), b(i)\big)}$$

where $a(i)$ is the average intra-cluster distance (the mean distance between a point and other points in the same cluster), and $b(i)$ is the lowest average inter-cluster distance (the mean distance between a point and points in the nearest neighboring cluster). The silhouette score ranges from 0 to 1, where higher values indicate better-defined clusters. An additional optimality criterion is the first local maximum.

Machine learning (ML) classification into mushroom, thin and stubby dendritic spines types was performed using SVM, LightGBM and XGboost classification models trained on manually labeled dataset.

1.4 Zernike moments for 2D images

Zernike moments are a set of polynomials above complex field that form orthonormal basis onto unit circle. In polar coordinates system the Zernike moment of radial order m and azimuthal order n, |n|≤m, is defined as:

$$V_{mn}(r, \theta) = R_{mn}(r)e^{in\theta}$$

Where $R_{mn}(r)$ is radial polynomials that are defined as following:

$$R_{mn}(r) = \sum_{s=0}^{m-|n|} (-1)^s \frac{(2m+1-s)!}{s!\,(m+|n|-s)!\,(m-|n|-s)!} r^{m-s}$$

The binary 2-dimensional object defined inside unit disk is then described by infinite sum:

$$f(r, \theta) = \sum_{m>0} \sum_{|n|\le m} a_{mn} V_{mn}(r, \theta)$$

The approximation of the original intensities on unit disk in polar coordinates can be obtained by selecting the maximum approximation order M. In this case, the approximation takes the following form:

$$f_M{}'(\theta, \phi) = \sum_{m\le M} \sum_{|n|\le m} a_{mn} V_{mn}(r, \theta)$$

To reconstruct approximate unit circles the algorithm of polar image generation was implemented and used (Xin, et al., 2005).

1.5 Spherical harmonics for 3D shapes

Spherical harmonics is the spherical function originating from solving Laplace's equation in the spherical domains. The principal characteristic of these functions is that they constitute an orthonormal basis in spherical coordinates. The value of spherical harmonic function is complex value. Each harmonic function is parametrized by polynomial degree (l) and order (m, |m| ≤ l):

$$Y_l^m(\theta, \phi) = e^{im\phi} N_l^m P_l^m(\cos\theta),$$

where *l* – harmonic degree, *m* – integer index, $P_l^m(\cos\theta)$ - associated Legendre polynomials, N_l^m — normalization factor, θ and ϕ— spherical coordinates, $e^{im\phi}$ — complex exponential term.

Complex spherical harmonics are invariant to the rotation of the input approximated spherical function f. However, reconstructing a surface from its decomposition into spherical functions requires the use of real-valued spherical harmonics. Real valued spherical harmonics functions are also orthonormal basis and could be used to approximate polygonal mesh object as a function in spherical coordinates. The equation for real valued spherical harmonics is as follows:

$$Y_l^m(\theta,\phi) = \frac{1}{\sqrt{2\pi}} e^{im\phi}\Theta_{lm}(\theta),$$

where l – harmonic degree, m – integer index ($|m| \le l$), θ and ϕ — spherical coordinates, $e^{im\phi}$ — complex exponential term, $\Theta_{lm}(\theta)$ – part of harmonic function, related from zenith angle and is expressed as:

$$\Theta_{lm}(\theta) = \sqrt{\frac{2l+1}{2}\frac{(l-m)!}{(l+m)!}} P_l^m(\cos\theta),$$

where $P_l^m(x)$ is is an associated Legendre polynomial and is expressed in terms of Legendre polynomials $P_n(z)$ by the formulas:

$$P_n(z) = \frac{1}{2^n n!}\frac{d^n}{dz^n}(z^2-1)^n$$

$$P_l^m(x) = \begin{cases} \frac{(l-|m|)!}{(l+|m|)!}(1-x^2)^{|m|/2}\frac{d^{|m|}}{dx^{|m|}}P_l(x), & m < 0 \\ (-1)^m(1-x^2)^{m/2}\frac{d^m}{dx^m}P_l(x), & m \ge 0 \end{cases}$$

The spherical object is then described by infinite sum:

$$f(\theta,\phi) = \sum_{l>0}\sum_{|m|\le l} a_{lm}Y_l^m(\theta,\phi)$$

The approximation of the original surface in spherical coordinates can be obtained by selecting the maximum polynomial degree L. In this case, the approximation takes the following form:

$$f_L{}'(\theta,\phi) = \sum_{l\le \mathrm{L}}\sum_{|m|\le l} a_{lm}Y_l^m(\theta,\phi)$$

1.6 Optimal hyper parameters estimation for Spherical Harmonics and Light Field

To determine the optimal parameter values for both descriptors, a systematic grid search was performed across the full range of integer combinations shown in Figure S2.

For the Spherical Harmonics (SH) descriptor, the approximation degree L was varied from 2 to 20 with a step of 1, and the number of sampled spherical points N was varied over the discrete set {5,10,20,30,40,50,60,80,100,140,180,200,240,260,280}. For each parameter combination, two convergence metrics were computed: the Hausdorff distance between the reconstructed and original surfaces, and the mean difference in decomposition coefficients between neighboring parameter configurations.

For the Light Field descriptor, the number of projection views was varied as {3,5,7,10}, where the minimum of three corresponds to orthogonal projections, and additional projections were obtained by varying angular offsets. The approximation order was varied from 1 to 19 with a step of 1. Convergence was assessed using the Procrustes distance between reconstructed shapes and the Spearman correlation between coefficient sets corresponding to neighboring parameter combinations.

To quantitatively define convergence, we considered that a parameter region reached a plateau when the relative change in both evaluation metrics between consecutive parameter increments fell below 5%. The optimal parameter region was thus defined as the smallest area where both the reconstruction accuracy (low Hausdorff or Procrustes distance) and coefficient stability (high Spearman correlation or minimal coefficient difference) simultaneously satisfied this convergence criterion.

Based on this analysis, the optimal parameters were found to be L=10 and N=140 for the Spherical Harmonics descriptor, and 5 projection views with an approximation order of 10 for the Light Field descriptor. These values represent the most efficient balance between reconstruction accuracy, coefficient stability, and computational complexity for the analyzed dataset

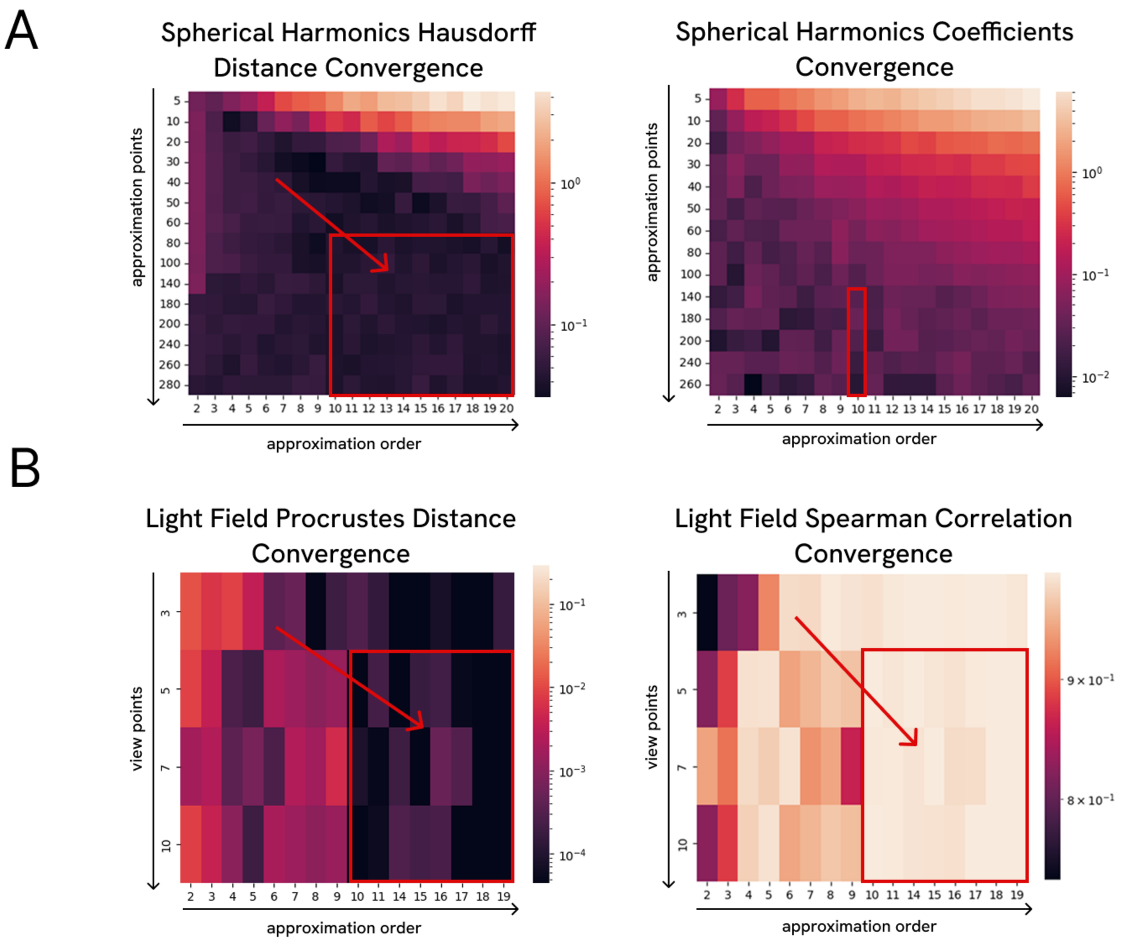

Figure S1. Optimal hyper parameters of novel complex shape descriptors greed search results.

A. Spherical Harmonics convergence graphs. Convergence graph of the approximation accuracy for the original surface, described with heatmap of Hausdorff distance values determines optimal approximation degree (obtained optimal decomposition degree is 10). While the convergence graph of the decomposition coefficient values presented with heatmap of difference in decomposition coefficients for neighbouring parameters. Graph allows for refining the number of approximation points at which coefficient stability is achieved (obtained optimal sampling points number equal to 140).

B. Heatmaps of two metrics, Procrustes distance and Spearman correlation, used to assess the convergence of decomposition coefficients across neighbouring parameters. Optimal number of projections to describe 3-dimensional morphology of spines within analysed dataset is 5 and optimal order of approximation is 10. Red rectangles highlight the regions where optimal values are achieved. The arrow indicates the direction of parameter optimization.

1.7 Statistical analysis and visualization

For metric distribution and Hausdorff distance distribution for ellipse and spherical harmonics statistical analysis was conducted with GraphPad Prism software. To determine if the distribution is normal or not, the Kolmogorov-Smirnov test was calculated. If the variances of the distributions were homogeneous, a Student's t-test was used for the comparison; if not, Welch's t-test was used. If in the result of Kolmogorov-Smirnov test distributions were not normal, Mann-Whitney test were used. Machine classification of the datasets into traditionally distinguished classes with Svm, Xgboost and lightgbm methods was performed using python public packages scikit-learn 1.2.2, lightgbm 4.1.0 and xgboost 2.0.3 respectively. Dimensionality reduction to three dimensions with PCA and t-SNE was performed using python package scikit-learn 1.2.2, with UMAP public python package umap. Three-dimensional plots with spines coordinates and relevant cluster color were generated with python module plotly.express. Representative spines images with the corresponding cluster color were visualized using MeshLab software. Pearson’s chi-squared test implemented into python package scipy 1.7.1 was used to compare clusters proportions between control and Aβ group to choose best performed clustering result for each descriptor (choice was provided between clustering results with different dimensional reduction and without dimensional reduction as well). For the same purpose and also for the clustering results evaluation and separability of the control/Aβ group in clusters comparison Agresti-Caffo independence test implemented in python module statsmodels 0.14.4 was used.

2. Dataset description

Primary mice hippocampal neurons were transfected with the pLV-eGFP plasmid for visualization and cultivated in vitro for 16-17 days (control group). A portion of these neurons was exposed to amyloid toxicity conditions (Aβ group) for 72 hours prior to fixation to model low amyloid synaptotoxicity (Ustinova, et al., 2024). Confocal high-resolution Z-stack images of the dendrites were captured and then deconvolved. As mentioned above, polygonal meshes of the dendrites were generated, and spines were segmented using SpineTool software.

Sixty-one Z-stack confocal images were obtained, 37 of them are images of control dendrites and 24 are dendrites under low-amyloid toxicity condition. With SpineTool Software 608 spine meshes were segmented, 300 are meshes of the control group and 308 are meshes of the spines under low-amyloid toxicity condition. During manual classification and after metrics calculation spines with invalid shapes or with segmentation defects (e.g. when metric ‘Junction area’ is equal zero) were excluded from the dataset. Eventually, 58 spines (9.5% of the all segmented spines) were excluded from analysis, leaving 279 spine meshes of the control group and 271 of the other in the dataset (550 overall). Moreover, for some of this 550 dendritic spines, it was impossible to calculate the spherical harmonic and Zernike

moment coefficients, therefore they were not used for clustering based on these descriptors. For Zernike moments, the clustering dataset comprised 527 spines (256 Aβ, 271 control), for spherical harmonics – 531 (259 Aβ, 272 control). For this reason, the subsection 4.4 also presents the clustering results based on classical metrics and chords on a reduced dataset consisting of the spines that were used in the Zernike moment clustering.

All spines were classified by 7 experts into 5 classes: mushroom, stubby, thin, filopodia and outliers. Outliers were excluded from the dataset prior to further analysis, as mentioned above. Manual classification results in each group were also compared using the Agresti-Caffo independence test, which allows analysing differences between two proportions (Fig. S1C). In the Aβ group, there are fewer mushroom spines (40.22%) compared to the control group (50.90%, $p = 0.012$). Mushroom spines are considered stable and are associated with long-term memory consolidation. Conversely, the proportion of thin spines is higher in the AD cell modeling group (25.83%) compared to the control group (17.92%, $p = 0.025$). Additionally, the proportion of filopodia spines, which do not form functional synapses (38), increases from 2.51% in the control group to 11.81% in the Aβ group ($p < 0.0001$). The data suggest that the shape of synapses is affected by the low-amyloid toxicity conditions, simulating AD in vitro.

Eleven scalar features were extracted for each spine, and distribution of these metrics within each experimental group were compared (S2A Fig). Control spines have statistically significant wider open angle (0.721° [0.606;0.841] and 0.659° [0.544;0.788], $p<0.0001$), larger Junction area (0.404 µm2 [0.251;0.631] and 0.317µm2 [0.186;0.496], $p<0.0001$) and Volume (0.479µm3 [0.277;0.870] and 0.418µm3 [0.210;0.744], $p=0.056$), than Aβ spines. Coefficient of variation in distance (CVD) (0.386±0.00528 for control and 0.401±0.00476 $p=0.0407$), length/volume (2.630 1/µm2 [1.814;3.812] and 3.408 1/µm2 [2.158;4.745], $p<0.0001$), length/area (0.305 1/µm [0.246;0.370] and 0.349 1/µm [0.278;0.417], $p<0.0001$) and convex hull ratios (0.373 [0.248;0.541] and 0.440 [0.260;0.696], $p=0.0037$) are lower in the control spines group rather than in AD model spines. Comparisons of four other scalar metrics (average distance, length, surface area, and convex hull volume) did not show significant differences. The Chord Length Distribution Histogram (CLDH), as proposed in (Pchitskaya, et al., 2023), was calculated for each spine. The mean distribution visual forms for each group (control and Aβ) and the differences between them are presented in Fig. S2B. Thus, the acquired dataset shows statistical differences between spine shapes in the control and Aβ groups, suggesting the need for further investigation to better distinguish clusters during clustering using both established and new descriptors.

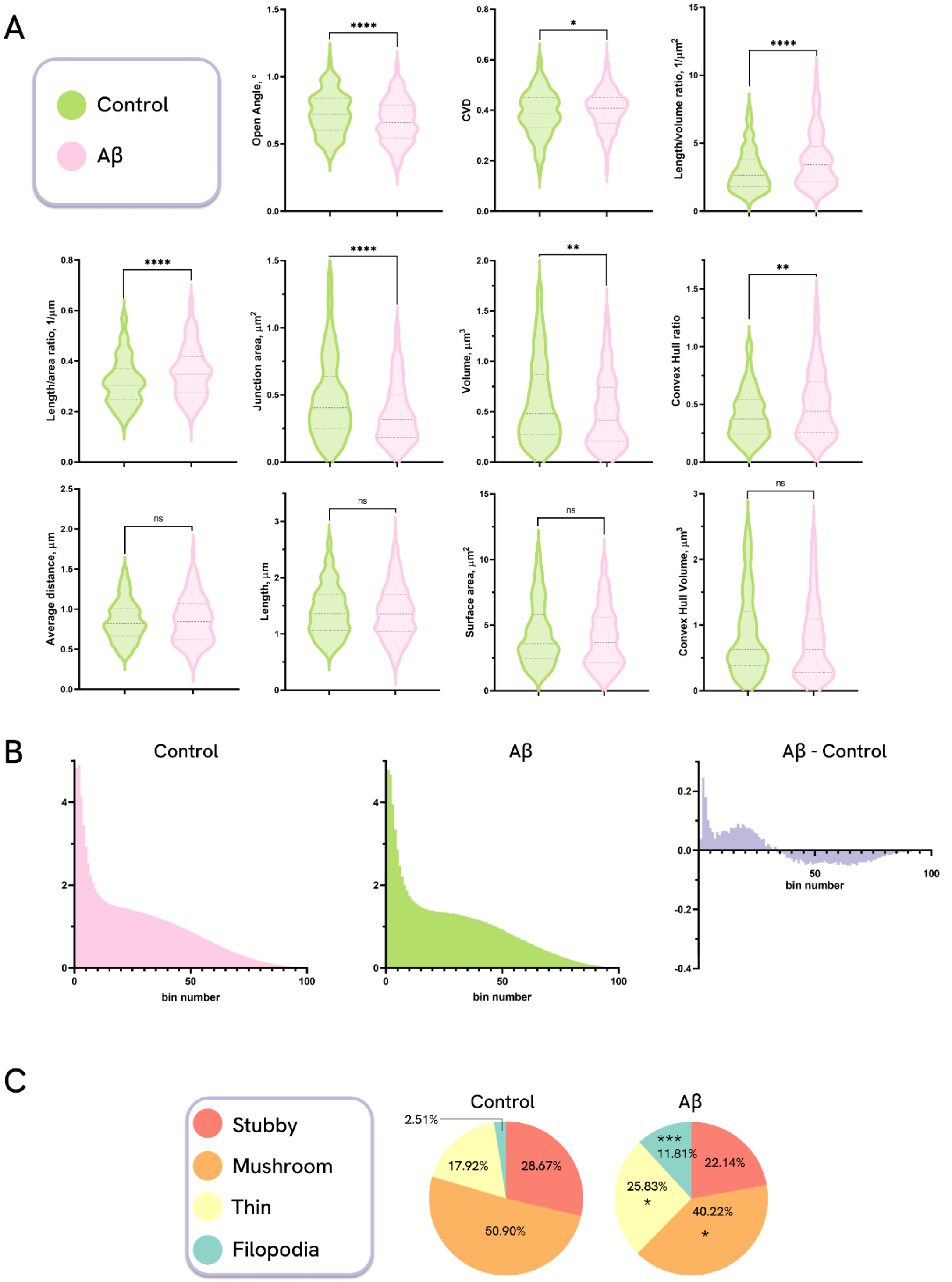

Figure S2. In vitro hippocampal dendritic spines dataset characteristics in norma and Alzheimer`s disease modeling low amyloid toxicity conditions.

A. Scalar metrics calculated using SpineTool software among the control and low amyloid toxicity conditions (Ab) (only statistically significant have shown). Coefficient of variation in distance (CVD) is shown for control and Aβ group as median with interquartile range [Q1;Q3] (Welch's t-test). Open angle, Length/volume ratio, length/area ratio, junction area, volume, convex hull ratio, length, average distance, area and convex hull volume are shown as median with interquartile range [Q1;Q3] (Mann-Whitney test). B. Mean chord length distribution histograms

calculated for each experimental group and difference between them. C. Classes distribution in control and Aβ group, obtained by experts classification (Agresti-Caffo independence test). ****:p<0.0001,***:p<0.001, *:p<0.05

3. Manual classification results shape approximation and ML classification

3.1 Spine classes approximation

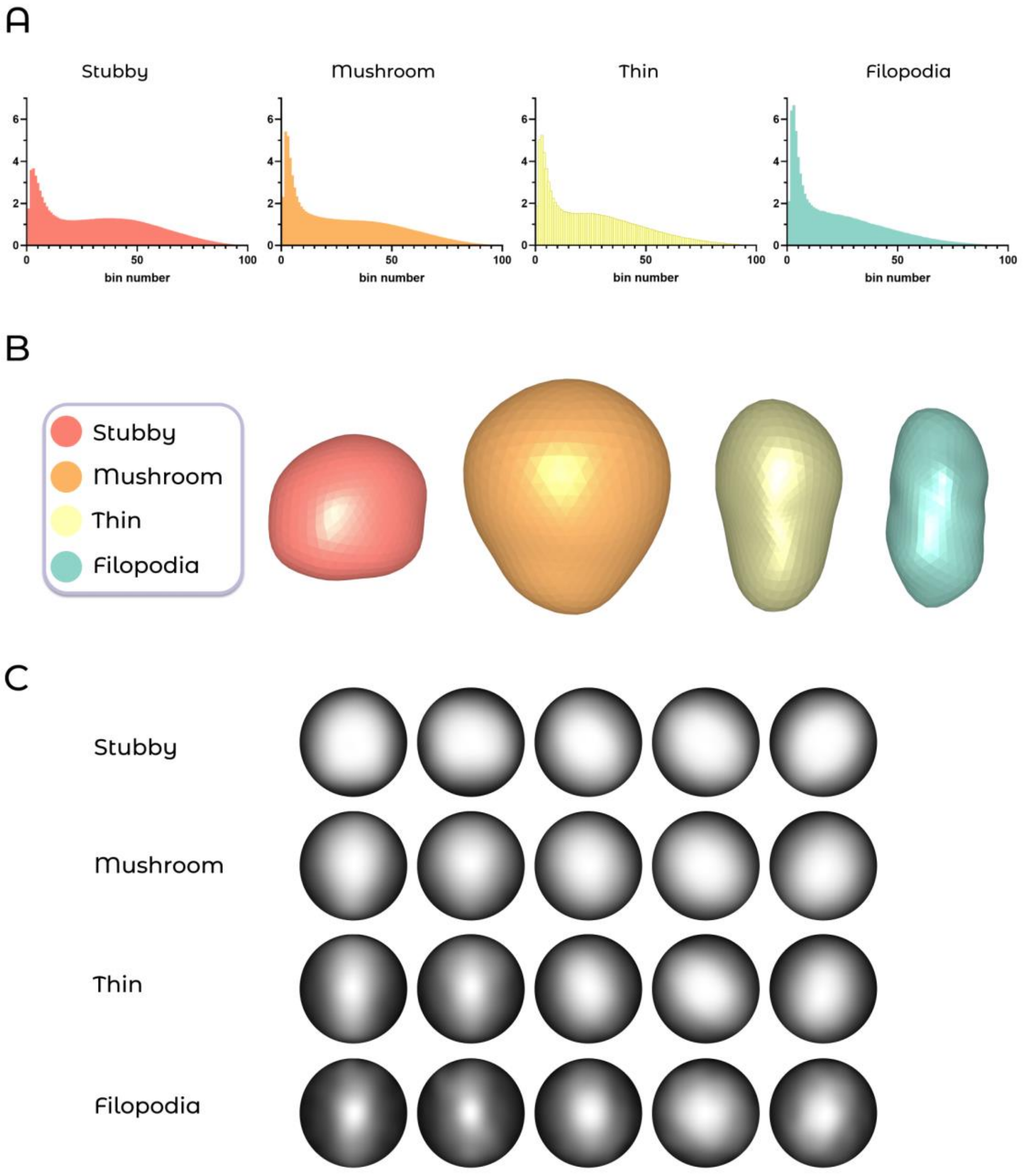

Figure S3. Mean representation of classical spine type classes for different complex shape descriptors.

A. Statistical representation of spine shape via mean of CHLD metric. B. 3-dimensional representation of mean shape of spine class via reconstruction of mean Spherical Harmonics decomposition coefficients. C. 2-dimensional representation of mean spine silhouette for different viewpoints obtained with reconstruction of mean Zernike moments decomposition coefficients for each observation point from Light Field descriptor.

3.2 ML spines classification using new 3D shape descriptors

The classical approach to grouping spines remains popular, so it is also important to test novel features in classification tasks. Various classification models (SVM, LightGBM and XGboost) were trained on two datasets—one from our previous research (Pchitskaya, et al., 2023) and the others from the current study—to assess accuracy and evaluate the performance of the newly proposed descriptors. Cross-validation was employed to obtain objective accuracy estimates; k-fold cross validation was performed for k=5. The classification accuracy results and manual markup accuracy are presented in Table S1.

The clustering experiment reveals systematic differences in performance across descriptor families. Manual classification is used as a reference point: *its accuracy reflects how often an individual expert's labels match the consensus label*, where the consensus is defined following the SpineTool procedure (Pchitskaya, et al., 2023).

Classical morphology metrics provide the highest and most stable accuracy across all classifiers, approaching the level of agreement observed between human annotators. Spherical Harmonics perform similarly well, showing accuracy values close to the classical baseline and demonstrating that harmonic decomposition effectively captures informative 3D shape variation of dendritic spines. Light Field modulus descriptors achieve intermediate accuracy, consistently outperforming CLDH but remaining below classical metrics and spherical harmonics. CLDH shows the lowest accuracy and stability across all models, indicating limited discriminative power in clustering task.

Overall, the new descriptors provide a more informative 3D representation than CLDH and achieve accuracy comparable to experimenter-level classification.

Table S1. Classification accuracy results.

Metric space	Classification method	SpineTool dataset accuracy	Current dataset accuracy
Classical 11 metrics	SVM	**0.77±0.5**	**0.79 ± 0.04**
	XGboost	0.72±0.07	0.70 ± 0.04
	LightGBM	0.75±0.05	0.75 ± 0.03
CLDH	SVM	0.55±0.06	0.68 ± 0.03
	XGboost	0.61±0.02	0.68 ± 0.02
	LightGBM	0.60±0.01	0.65 ± 0.02
Spherical Harmonics	SVM	0.75±0.7	0.73 ± 0.02
	XGboost	0.69±0.08	**0.79 ± 0.03**
	LightGBM	**0.77±0.2**	0.75 ± 0.03

Light Field modulus	SVM	0.69±0.05	0.70 ± 0.03
	XGboost	0.76±0.7	0.73 ± 0.03
	LightGBM	0.73±0.05	0.71 ± 0.02
Manual classification		**0.77±0.06**	**0.82 ± 0.07**

4. Clustering results information

4.1 Evaluating numbers of clusters

For K-means clustering, the number of clusters is not chosen automatically. Therefore, for each method, elbow and silhouette methods were applied (FigS4 and Fig S5). The elbow method determines the optimal number of clusters at the inflection point of the graph, while the silhouette method uses the first local maximum or the global maximum.

For our dataset, the silhouette scores were relatively low and local maxima were not always present. For this reason, the elbow method was the primary technique for selecting the number of clusters. Since the elbow method is partially subjective regarding the selection of the inflection point, in cases where we observed several potential inflection points, several cluster number options were selected using this metric. The primary biological relation was that there should be at least four clusters, as this matches the number of classes in the traditional classification. On average, the results from the silhouette and elbow methods indicate cluster numbers ranging from 4 to 8, most frequently 5-7. We also relied on previous studies where k=5 was chosen for clustering of dendritic spines, including those on larger datasets (Ferreira, et al.) . We hypothesize that as the number of polygonal meshes of dendritic spines in the dataset increases, the results of the silhouette and elbow scores will converge to 1-2 specific values.

.

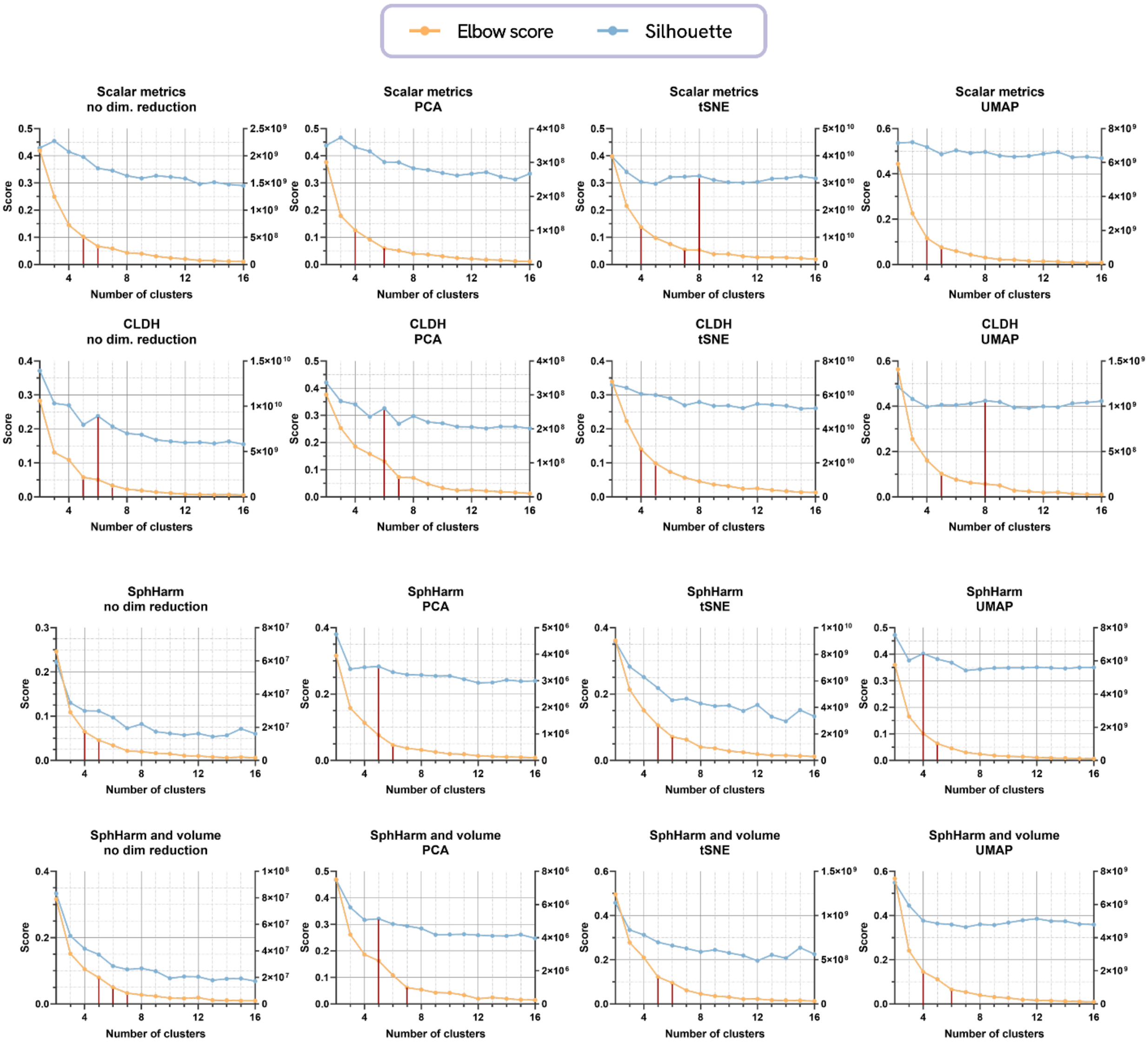

Figure S4. Number of clusters selection methods score graphs for each clusterization based on scalar metrics, Chord length distribution (CLDH) and spherical harmonics (SphHarm). Left Y-axis is for silhouette. Right Y-axis is for elbow score. Red vertical lines show chosen numbers of clusters.

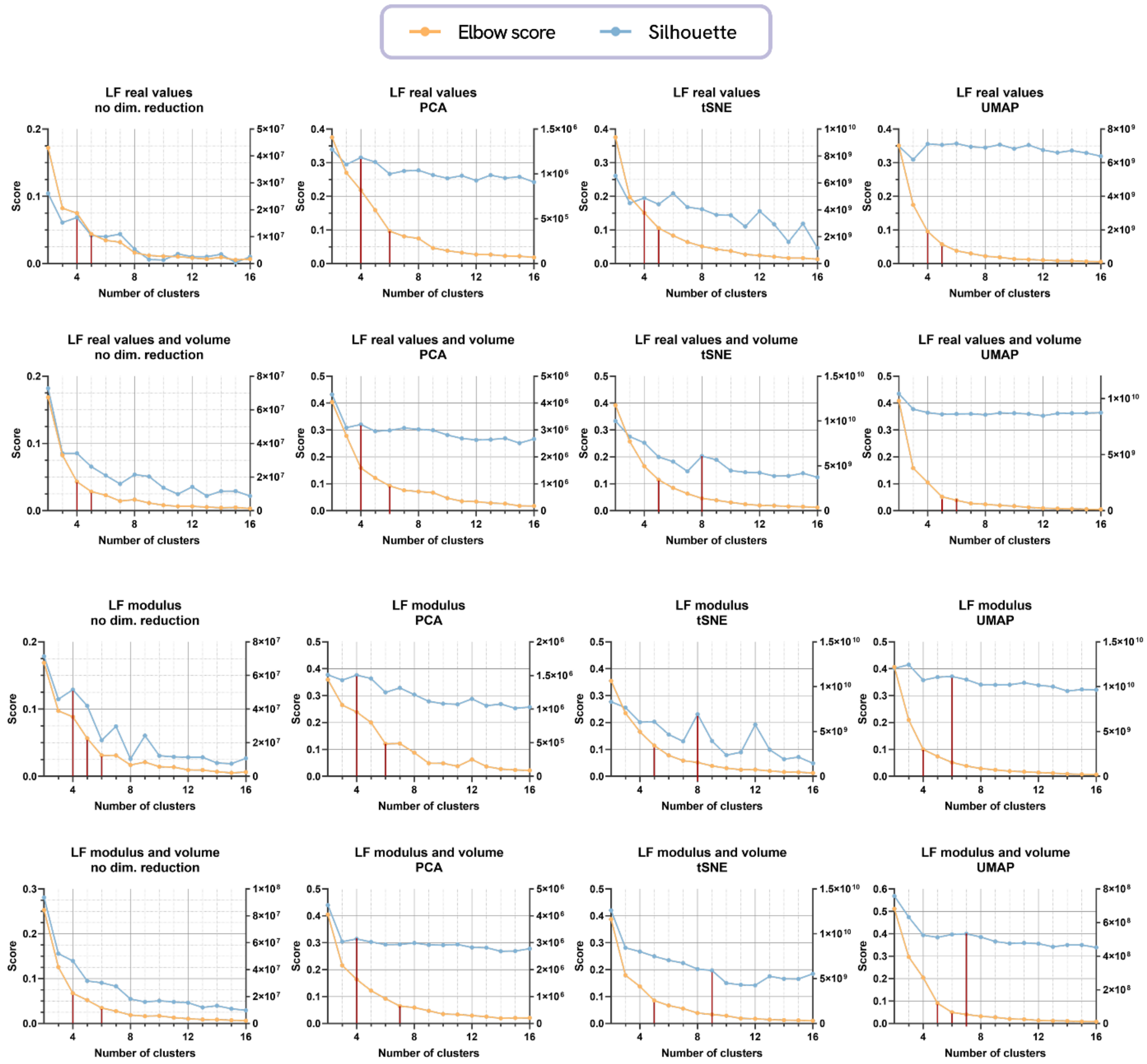

Figure S5. Number of clusters selection methods score graphs for each clusterization based on Zernike moments (Light Field). Left Y-axis is for silhouette. Right Y-axis is for elbow score. Red vertical lines show chosen numbers of clusters.

4.2 Comparison of clustering results for different metric spaces

Each morphological descriptor defines its own metric space, within which the clustering of dendritic spines was performed. These spaces are denoted as classic, chord, light field, and spherical harmonics. Since all examined feature spaces are high-dimensional, several dimensionality reduction techniques—PCA, UMAP, and t-SNE—were additionally tested for each of them. The quality of clustering was assessed by evaluating the degree of separation between dendritic spines from control cultures and those exposed to amyloid toxicity. Two statistical measures were employed for this purpose: the Agresti–Caffo independency test, that shows differences of groups in each cluster, and the two-sample χ^2 test, that shows difference between distributions of two groups (Table S2). Based on these metrics, an optimal

dimensionality reduction method and best performance was determined for each descriptor (best perfomances are highlighted bold in Table S2, FigS6 and FigS7 are for new descriptors).

Table S2. Clustering separability of all descriptors for various dimensionality reduction methods and optimal *k*. The best results for separability are highlighted in bold.

Shape descriptor	Dimension	Number of clusters	How many clusters are statistically different (p-value of Agresti-Caffo test >0.05)	What are p-values of Agresti-Caffo for this clusters	Chi-square
Classic	No dim. reduction	5	0	-	0.129
	No dim. reduction	6	1	0.0402	0.0454
	PCA 3 dim.	4	1	0.0473	0.0436
	PCA 3 dim.	6	0	-	0.0521
	tSNE 3 dim.	4	1	0.0667	0.0455
	**tSNE 3 dim.**	**7**	**2**	**0.0370; 0.000802**	**0.00390**
	tSNE 3 dim.	8	2	0.0370; 0.00102	0.00663
	UMAP 3 dim.	4	1	0.00104	0.0109
	UMAP 3 dim.	5	2	0.0272; 0.00624	0.0126
Chord	No dim. reduction	5	0	-	0.322
	No dim. reduction	6	0	-	0.247
	**No dim. reduction**	**7**	**1**	**0.0206**	**0.201**
	PCA 3 dim.	6	0	-	0.288
	**PCA 3 dim.**	**7**	**1**	**0.0373**	**0.282**
	tSNE 3 dim.	4	0	-	0.416
	tSNE 3 dim.	5	0	-	0.468
	UMAP 3 dim.	5	0	-	0.347
	UMAP 3 dim.	8	0	-	0.862
SphHarm	No dim. reduction	4	2	0.00608; 0.00827	0.00940
	No dim. reduction	5	2	0.0303; 0.0128	0.00889
	**PCA 3 dim.**	**5**	**3**	**0.0473; 0.00161; 0.0473**	**0.00262**
	PCA 3 dim.	6	2	0.00478; 0.00272	0.000492
	tSNE 3 dim.	5	2	0.00332; 0.000589	0.00266
	tSNE 3 dim.	6	2	0.0241; 0.0136	0.0181
	UMAP 3 dim.	4	2	0.0178; 0.00175	0.0108
	UMAP 3 dim.	5	2	0.0407; 0.0113	0.00153
SphHarm + Volume	No dim. reduction	5	1	0.00644	0.0602
	No dim. reduction	6	2	0.0463; 0.0432	0.0931
	No dim. reduction	7	2	0.0365; 0.0121	0.0836
	PCA 3 dim.	5	1	0.00549	0.0294
	PCA 3 dim.	7	2	0.0308; 0.0151	0.0393
	tSNE 3 dim.	5	2	0.0238; 0.00443	0.0138
	tSNE 3 dim.	6	3	0.0402; 0.00886; 0.000216	0.000966
	UMAP 3 dim.	4	2	0.0128; 0.00767	0.0114
	**UMAP 3 dim.**	**6**	**4**	0.0311; 0.0203; 0.00705; 0.00607	**0.000915**
LightField real values	No dim. reduction	4	2	0.00270; 0.00380	0.0130
	No dim. reduction	5	1	0.0243	0.104
	PCA 3 dim.	4	2	0.0134; 0.0335	0.0698
	**PCA 3 dim.**	**6**	**3**	**0.0186; 0.0363; 0.0339**	**0.0114**

	tSNE 3 dim.	4	1	0.000854	0.00895
	tSNE 3 dim.	5	2	0.0132; 0.00000461	0.000123
	UMAP 3 dim.	4	2	0.00462; 0.000870	0.00270
	UMAP 3 dim.	5	2	0.00812; 0.0000286	0.000213
LightField real values + Volume	**No dim. reduction**	**4**	**3**	**0.0471; 0.00704; 0.0000913**	**0.000109**
	No dim. reduction	5	2	0.00622; 0.00954	0.0103
	PCA 3 dim.	4	3	0.0176; 0.0442; 0.0000843	0.000231
	PCA 3 dim.	6	2	0.0380; 0.0181	0.0566
	tSNE 3 dim.	5	1	0.00778	0.0515
	tSNE 3 dim.	8	2	0.0350; 0.00425	0.0278
	UMAP 3 dim.	5	2	0.0176; 0.00653	0.0272
	UMAP 3 dim.	6	2	0.0245; 0.00445	0.0134
LightField modulus	No dim. reduction	4	2	0.0441; 0.000176	0.000195
	**No dim. reduction**	**5**	**4**	**0.0432; 0.0446; 0.000116; 0.000811**	**0.00000331**
	No dim. reduction	6	2	0.000504; 0.000312	0.0000233
	PCA 3 dim.	4	3	0.0118; 0.0186; 0.00103	0.000185
	PCA 3 dim.	6	3	0.000823; 0.000525	0.0000419
	tSNE 3 dim.	5	2	0.0164; 0.00622	0.00340
	tSNEn 3 dim	8	2	0.00509; 0.0000936	0.000558
	UMAP 3 dim.	4	1	0.000417	0.00566
	UMAP 3 dim.	6	2	0.0341; 0.00000746	0.0000974
LightField modulus + Volume	No dim. reduction	4	2	0.00441; 0.000922	0.000856
	No dim. reduction	6	1	0.0000715	0.000936
	PCA 3 dim.	4	2	0.0104; 0.0144	0.0121
	PCA 3 dim.	7	1	0.00000302	0.0000943
	tSNE 3 dim.	5	2	0.0477; 0.0295	0.0261
	tSNE 3 dim.	9	3	0.0124; 0.0165; 0.0000126	0.0000511
	**UMAP 3 dim.**	**5**	**2**	**0.00647; 0.000147**	**0.000294**
	UMAP 3 dim.	6	1	0.000582;	0.0128
	UMAP 3 dim.	7	2	0.0189; 0.000582	0.00770

For most descriptors, a clear improvement in clustering quality was observed when dimensionality was reduced to three components. This effect is likely related to the limited size of the dataset and the dispersion of points in the original high-dimensional spaces. An exception was observed for the light field descriptor, where dimensionality reduction either yielded results comparable to the original or led to a reduction in group separability. This indicates that important information essential for distinguishing morphologies is lost in lower dimensions. Consequently, light field descriptor appears to have lower feature redundancy for the given dataset.

Light field descriptor with absolute values and without dimensionality reduction demonstrates the best results among all descriptors (Table Sk). It shows the highest number of clusters with significant Agresti–Caffo statistics (4 clusters: 2 with $p<0.05$ and 2 with $p<0.001$) and the strongest χ^2 test significance ($p<0.00001$). The inclusion of the volume metric into this descriptor substantially alters the structure of the metric space and reduces the separability of morphological clusters with respect to both statistical measures. However, applying dimensionality reduction in this case partially compensates for this effect, leading to improved clustering results (2 Agresti-Cafo clusters: 1 with $p<0.01$ and 1 with $p<0.001$; χ^2 with

$p<0.001$). In contrast, for light field descriptor with real-valued features, the inclusion of the volume metric also affects the structure of the space but improves group separability, rendering dimensionality reduction unnecessary.

For spherical harmonics descriptor, the inclusion of the volume metric does not cause substantial changes in the structure of the space. The positive effect of dimensionality reduction is preserved, and the number of significant clusters according to the Agresti–Caffo statistic increases by one (with $p<0.01$) compared to the baseline configuration without volume values.

In the case of classic metrics, the application of dimensionality reduction consistently improves clustering results across all tested methods, indicating that this descriptor retains its discriminative power even after feature compression.

In contrast, chords descriptor shows no noticeable improvement: χ^2 statistics remain insignificant both before ($p=0.2$) and after ($p=0.28$) dimensionality reduction, suggesting that the metric space formed by this descriptor does not effectively separate control and Aβ dendritic spines.

Overall, the analysis indicates that the influence of dimensionality reduction methods depends strongly on the internal structure and redundancy of the feature sets defined by each descriptor. For most descriptors, reducing dimensionality enhances the formation of distinct morphological clusters, whereas in specific cases, such as light field descriptor, it can result in the loss of discriminative information.

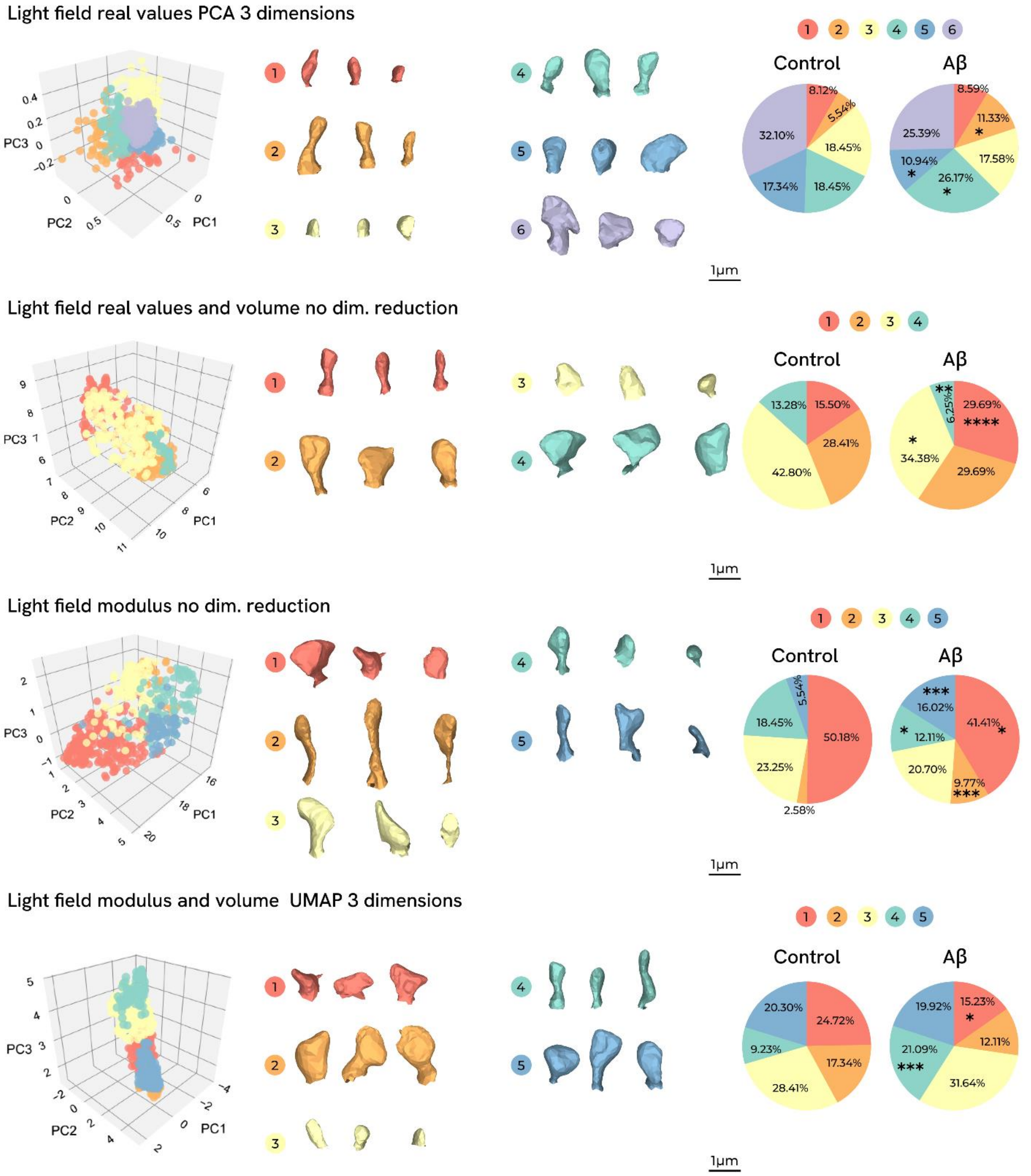

Figure S6. Spine clustering results for each Light Field clustering. On the left side of the figure — spines clustering maps with every metric in three-dimensional coordinates using UMAP reduction method. Three-dimensional plots codes are presented in the supplementary data. In the middle - representative spines for each cluster. On the right side of the figure — pie chart representation of the cluster distribution in the control and experimental group. For the control/Aβ comparison Agresti-Caffo independence test used. Light Field real values metric space was dimensionally reduced to three dimensions with UMAP before clustering, others clustering procedures were performed without dimensional reduction. ****:p<0.0001, ***:p<0.001, **:p<0.01, *:p<0.05

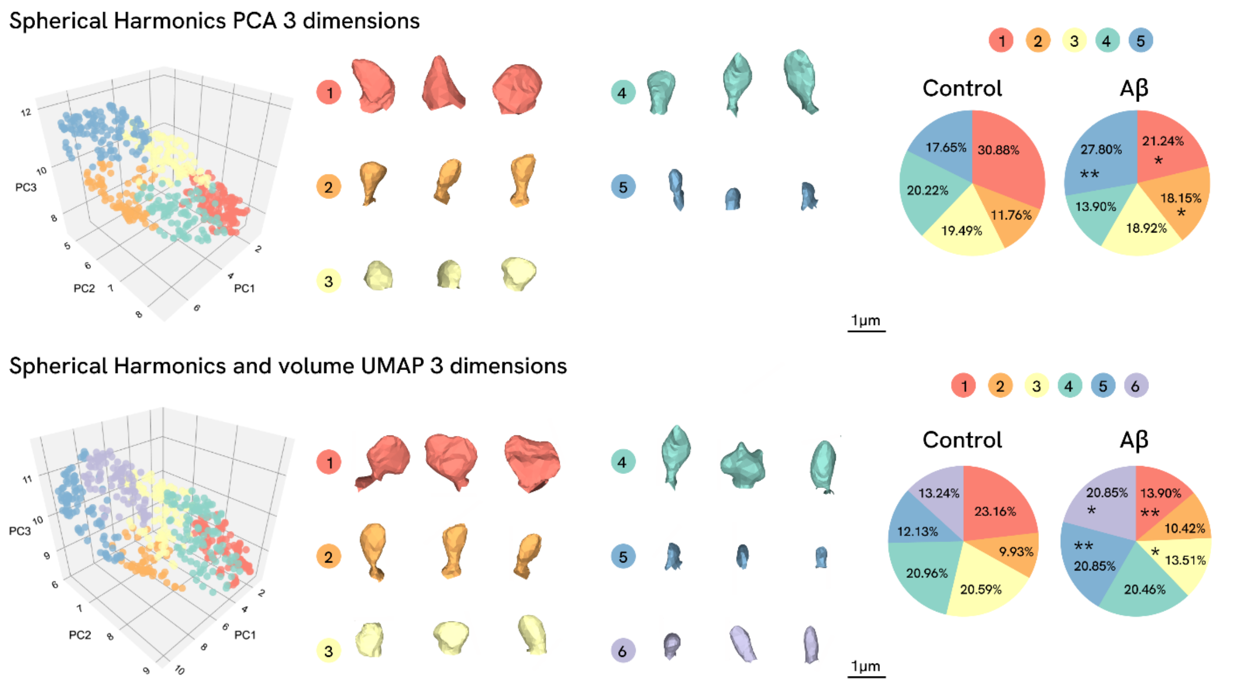

Figure S7. Spine clustering results for each Spherical Harmonics clustering. On the left side of the figure — spines clustering maps with every metric in three-dimensional coordinates using UMAP reduction method. Three-dimensional plots codes are presented in the supplementary data. In the middle - representative spines for each cluster. On the right side of the figure — pie chart representation of the cluster distribution in the control and experimental group. For the Control/Aβ comparison Agresti-Caffo independence test used. Both descriptors space was dimensionally reduced to three dimensions with UMAP before clustering.**:p<0.01, *:p<0.05

4.3 Clusters separability validation through control–control comparison

To validate the results of morphological clustering distinguishing control and Aβ dendritic spines, an additional experiment was conducted using only dendritic spines from wild-type mouse cultures. A subset of 279 control dendritic spines was extracted from the dataset for this purpose.

Clustering was performed in the metric spaces corresponding to the descriptors and dimensionality reduction methods that had shown the best performance in the main analysis: t-SNE (3D) for classic metrics descriptor, PCA (3D) for chords descriptor, UMAP (3D) for spherical harmonics descriptor extended with the volume metric, and no dimensionality reduction for light field descriptor with absolute values. The number of clusters, k, varied from 4 to 8, matching the range of morphological group counts previously analyzed on the full dataset, score graphs are presented in figS8.

For each resulting clustering, dendritic spines were randomly divided into two artificial groups, simulating “Control 1” and “Control 2” classes. For each such division, Agresti–Caffo and two-sample χ^2 statistics were calculated, analogous to the procedure used to assess separability between control and Aβ gropus in the main dataset. This randomization procedure was repeated 100 times to obtain averaged results.

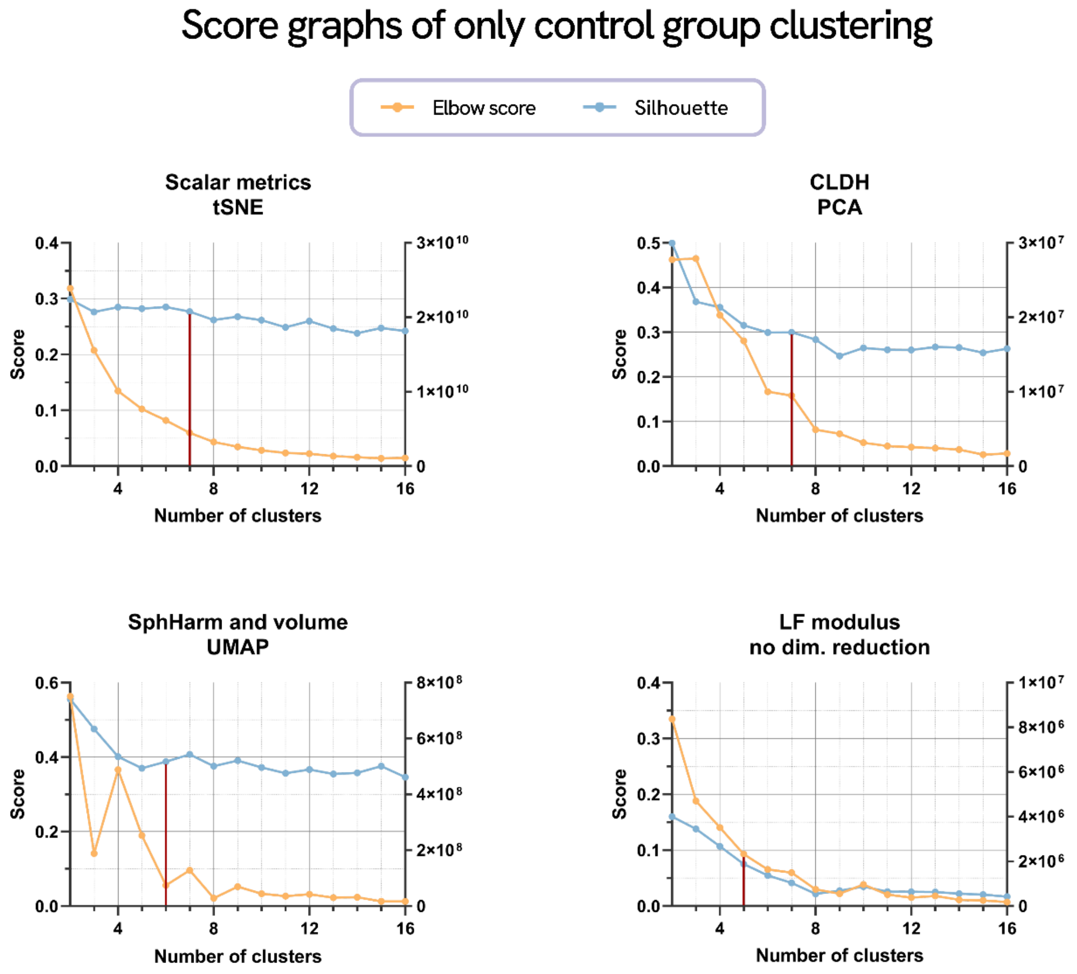

Figure S8. Number of clusters selection methods score graphs for each control-control clusterization. Left Y-axis is for silhouette. Right Y-axis is for elbow score. Red vertical lines show numbers of clusters that were chosen in the main clustering experiment.

Across all descriptors, the proportion of clusters that showed statistical significance in the Agresti–Caffo test with $p < 0.05$ did not exceed 5.8%, and with $p < 0.01$ did not exceed 1.38%. Similarly, the proportion of experiments with statistically significant χ^2 test results did not exceed 8% at $p < 0.05$ and 3% at $p < 0.01$. The most stable validation results at the $p < 0.01$ level were obtained for light field descriptor, where the proportion of significant results for both Agresti-Caffo and χ^2 tests did not exceed 1%. Notably, this descriptor also demonstrated the highest separability between control and Aβ groups in the main dataset (Table S3).

Table S3. Control–control validation: proportion of significant clusters across descriptors

Descriptor	Number of the cluster	Agresti-Caffo		Chi-square	
		% p-value > 0.05	% p-value <0.01	% p-value > 0.05	% p-value <0.01
Classic tSNE	4	94.25	0.5	96	0
	5	95.6	1.2	92	1
	6	95.83	1	94	1
	7	96.43	1	96	0
	8	95.125	0.75	98	0
Chord PCA	4	97	0.75	97	0
	5	97.6	0.6	97	1
	6	96.67	0.33	94	0
	7	94.72	0.71	92	2
	8	95.25	1.375	94	2
Lf modulus	4	96.25	0.5	98	0
	5	94.2	1	94	0
	6	96.67	1	92	1
	7	95.43	0.57	99	0
	8	96.375	1	97	0
SphHarm	4	97.75	1	98	1
	5	95.2	1.2	96	2
	6	95.5	0.5	94	0
	7	95.29	1.28	93	3
	8	95.125	0.5	93	1

4.4 Clustering results on scalar and CLDH metrics using reduced spines dataset

For the spherical harmonics and Zernike moments, a slightly smaller dataset of spines was used for clustering compared to the clustering based on scalar metrics and chords. This reduction in the dataset size could have affected the cluster separability results, potentially either improving or worsening them relative to the main dataset (550 spines). Therefore, we also clustered the Zernike moment dataset version (527 spines) using scalar metrics and chord distributions. The results are presented in Table S4. The Agresti-Caffo and Chi-square test values generally do not differ from the initial results for 550 spines dataset, which supports the validity of comparing the main clustering perfomances.

Table S4. Clustering separability of baseine descriptors for various dimensionality reduction methods and optimal *k* on reduced dataset.

Shape descriptor	Dimension	Number of clusters	How many clusters are statistically different (p-value of Agresti-Caffo test >0.05)	What are p-values of Agresti-Caffo for this clusters	Chi-square
Classic	No dim. reduction	4	1	0.0411	0.0315
	No dim. reduction	5	0	-	0.0859
	PCA 3 dim.	4	1	0.0411	0.0315
	PCA 3 dim.	5	0	-	0.111
	tSNE 3 dim.	5	2	0.00506; 0.000291	0.000196
	tSNE 3 dim.	7	2	0.0386; 0.00279	0.0104
	tSNE 3 dim.	8	1	0.00108	0.00880
	UMAP 3 dim.	4	1	0.00293	0.0300
	UMAP 3 dim.	5	2	0.01245; 0.0456	0.0267

Chord	No dim. reduction	5	0	-	0.378
	No dim. reduction	6	0	-	0.377
	PCA 3 dim.	6	0	-	0.329
	PCA 3 dim.	7	1	0.00750	0.0685
	tSNE 3 dim.	5	0	-	0.689
	tSNE 3 dim.	6	0	-	0.856
	UMAP 3 dim.	5	0	-	0.431
	UMAP 3 dim.	8	0	-	0.792

5. Reference

Ferreira, A., *et al.* Distinct forms of structural plasticity of adult-born interneuron spines in the mouse olfactory bulb induced by different odor learning paradigms. *Communications Biology* 2024;7(1):420.
Likas, A., Vlassis, N. and J. Verbeek, J. The global k-means clustering algorithm. *Pattern Recognition* 2003;36:451-461.

Pchitskaya, E., *et al.* SpineTool is an open-source software for analysis of morphology of dendritic spines. *Scientific Reports* 2023;13(1):10561.

Popugaeva, E., *et al.* STIM2 protects hippocampal mushroom spines from amyloid synaptotoxicity. *Molecular Neurodegeneration* 2015;10(1):37-37.

Sage, D., *et al.* DeconvolutionLab2: An open-source software for deconvolution microscopy. In, *Methods*. Academic Press Inc.; 2017. p. 28-41.

Sun, S., *et al.* Reduced Synaptic STIM2 Expression and Impaired Store-Operated Calcium Entry Cause Destabilization of Mature Spines in Mutant Presenilin Mice. *Neuron* 2014;82(1):79-93.

Ustinova, A., *et al.* Generate and Analyze Three-Dimensional Dendritic Spine Morphology Datasets With SpineTool Software. *Current Protocols* 2024;4(12):e70061-e70061.

Xin, Y., Pawlak, M. and Liao, S. Image reconstruction with polar Zernike moments. In, *Lecture Notes in Computer Science*. Springer Verlag; 2005. p. 394-403.
